# Structures of β_1_-adrenergic receptor in complex with Gs and ligands of different efficacies

**DOI:** 10.1038/s41467-022-31823-1

**Published:** 2022-07-14

**Authors:** Minfei Su, Navid Paknejad, Lan Zhu, Jinan Wang, Hung Nguyen Do, Yinglong Miao, Wei Liu, Richard K. Hite, Xin-Yun Huang

**Affiliations:** 1 Department of Physiology and Biophysics, Weill Cornell Medical College of Cornell University, New York, NY 10065 USA; 2 Structural Biology Program, Memorial Sloan Kettering Cancer Center, New York, NY 10065 USA; 3 Cancer Center and Department of Pharmacology and Toxicology, Medical College of Wisconsin, Milwaukee, WI 53226 USA; 4 Center for Computational Biology and Department of Molecular Biosciences, University of Kansas, Lawrence, KS 66047 USA

**Keywords:** Cryoelectron microscopy, Hormone receptors, G protein-coupled receptors

## Abstract

G-protein-coupled receptors (GPCRs) receive signals from ligands with different efficacies, and transduce to heterotrimeric G-proteins to generate different degrees of physiological responses. Previous studies revealed how ligands with different efficacies activate GPCRs. Here, we investigate how a GPCR activates G-proteins upon binding ligands with different efficacies. We report the cryo-EM structures of β_1_-adrenergic receptor (β_1_-AR) in complex with Gs (Gα_s_Gβ_1_Gγ_2_) and a partial agonist or a very weak partial agonist, and compare them to the β_1_-AR–Gs structure in complex with a full agonist. Analyses reveal similar overall complex architecture, with local conformational differences. Cellular functional studies with mutations of β_1_-AR residues show effects on the cellular signaling from β_1_-AR to the cAMP response initiated by the three different ligands, with residue-specific functional differences. Biochemical investigations uncover that the intermediate state complex comprising β_1_-AR and nucleotide-free Gs is more stable when binding a full agonist than a partial agonist. Molecular dynamics simulations support the local conformational flexibilities and different stabilities among the three complexes. These data provide insights into the ligand efficacy in the activation of GPCRs and G-proteins.

## Abstract (web summary)

Su et al. report cryo-EM structures of β_1_-adrenergic receptor, Gs and ligands of different efficacies. These complexes have similar overall architecture, but with local conformational differences and different stabilities.

## Introduction

G-protein-coupled receptors (GPCRs) mediate transmembrane signaling from ligands with different efficacies to downstream heterotrimeric G-proteins^1–5^. Ligands can vary in efficacy, namely in their intrinsic ability to activate downstream signaling pathways. Full agonists elicit the maximal signaling response, and partial agonists induce various degrees of sub-maximal responses. Antagonists produce no or little responses by themselves, but block the binding of other ligands to GPCRs. Inverse agonists decrease the basal physiological activity of GPCRs^1,5,6^. A conformation selection model has been proposed to explain the actions of ligands with different efficacies on GPCRs^4,5,7^. GPCRs are highly dynamic proteins and can sample multiple conformations including inactive states, intermediate states, and active states. These different conformations are in equilibrium with each other. Ligands stabilize unique and ligand-specific GPCR conformations. Binding of full agonists stabilizes the active state conformation, and shifts the GPCR conformational equilibrium towards the active states. Partial agonists select a different conformation and are less able to drive the equilibrium to the active state than full agonists. Therefore, the population or amount of GPCRs in the active state is correlated with ligand efficacy^4,5,7^.

After ligand binding, GPCRs activate G-proteins to initiate downstream physiological responses. Here we have investigated the activation of G-proteins by GPCRs bound with ligands with different efficacies. Previous X-ray crystal structural studies of GPCRs (without G-proteins) bound with ligands with different efficacies surprisingly showed similar conformations for individual GPCRs that most are in the inactive states^4^. The ligand-binding pockets in the receptors adopt ligand-specific configurations. On the other hand, nuclear magnetic resonance and fluorescence life-time spectroscopy studies of GPCRs (without G-proteins) indicate that ligand efficacy correlates with local conformational changes, and these changes occur in a fast timescale^8–17^. Furthermore, X-ray crystal and cryo-EM structures of the complexes of full agonist-bound GPCRs and G-proteins show that GPCRs in these complexes are in the fully active states^18,19^. While the interactions between GPCRs (without G-proteins) and full agonists, partial agonists and antagonists have been investigated, we still do not fully understand the structural and biochemical bases for the activation of G-proteins by GPCRs after bound with partial agonists^18,19^.

In this work, we use structural, computational, cellular and biochemical approaches to understand the mechanisms of activation of G-proteins by β_1_-adrenergic receptor (β_1_-AR) after bound with ligands of different efficacies. We determine the cryo-EM structures of β_1_-AR and heterotrimeric Gs (Gα_s_Gβ_1_Gγ_2_) in complex with a partial agonist (dobutamine), or a very weak partial agonist (cyanopindolol; also called an antagonist). We then compare these cryo-EM structures with our previously determined cryo-EM structure of β_1_-AR and Gs in complex with a full agonist (isoproterenol)^20^. This provides the opportunity to compare and contrast the interactions between the same GPCR and the G-protein in the presence of a full agonist, a partial agonist, and a very weak partial agonist. The comparison surprisingly reveals that the overall structures of the three different complexes are similar, with local conformational differences mainly in the ligand-binding pockets. Furthermore, we examine the activation of Gs by β_1_-AR in cells after stimulation by these three ligands. We generate mutations in the residues on β_1_-AR that interact with the ligands or Gs. These mutations impair the cellular signaling from β_1_-AR to the downstream cAMP response initiated by the three different ligands, with residue-specific differences. Moreover, we investigate the stability of the intermediate state complex of the nucleotide-exchange process (i.e. the ligand–β_1_-AR–nucleotide-free Gs complex). We find that, when bound with a full agonist, the β_1_-AR–Gs (nucleotide-free) intermediate state is more stable than the intermediate states bound with a partial agonist. All-atom simulations using a robust Gaussian accelerated molecular dynamics (GaMD) method^21,22^ support these structural and biochemical findings. Together, these data provide insights into the activation of G-proteins by GPCRs after bound with ligands of different efficacies.

## Results

### Cryo-EM structures of the complexes of Gs and β_1_-AR bound with a partial agonist or a very weak partial agonist

**Fig. 1:**
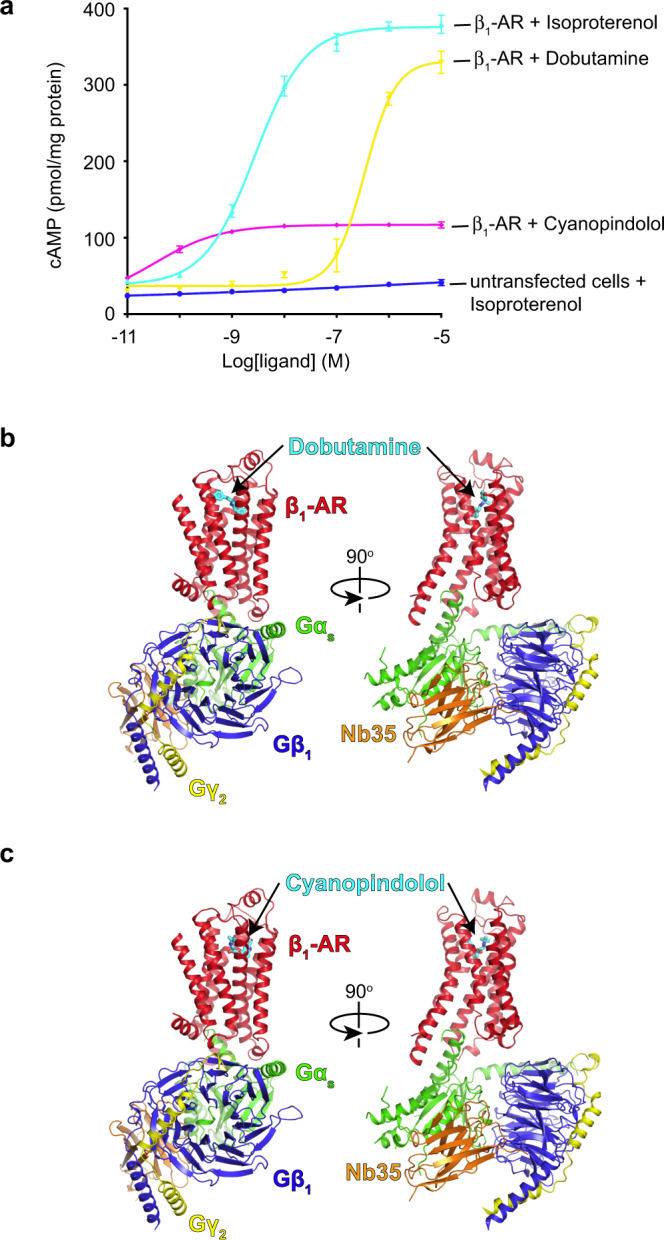
Cryo-EM structures of the complexes of Gs and β_1_-AR bound with a partial agonist or a very weak partial agonist. **a** Comparison of the cellular cAMP responses induced by isoproterenol, dobutamine, and cyanopindolol. Data are presented as mean ± SD of three experiments. Source data are provided as a Source Data file. **b** Cartoon diagrams of the dobutamine–β_1_-AR–Gs complex are shown. **c** Cartoon diagrams of the cyanopindolol–β_1_-AR–Gs complex are shown. β_1_-AR in red, Ras-like GTPase domain of Gα_s_ in green, Gβ in blue, Gγ in yellow, and Nb35 nanobody in orange.

**Fig. 2:**
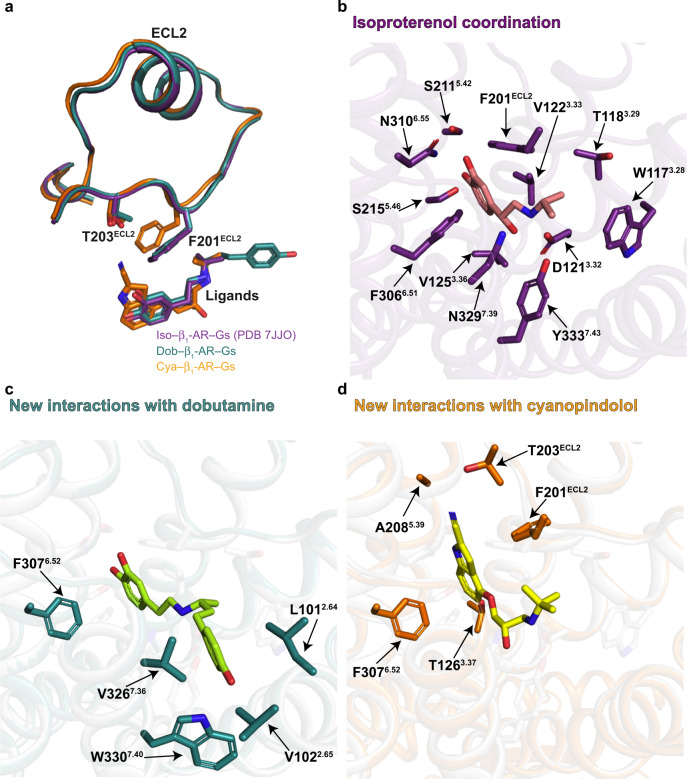
Local conformational differences among the three complexes. **a** ECL2 segments from the three complexes are shown. While Phe201^ECL2^ is involved in the binding to all three ligands, Thr203^ECL2^ is only engaged in cyanopindolol interaction. The color codes: Purple: Iso–β_1_-AR–Gs; Green: Dob–β_1_-AR–Gs; Orange: Cya–β_1_-AR–Gs. **b** Diagram of the ligand-binding residues in the isoproterenol-bound β_1_-AR–Gs complex. **c** Additional ligand-binding residues in the dobutamine-bound β_1_-AR–Gs complex, comparing with the isoproterenol-bound β_1_-AR–Gs complex. **d** Additional ligand-binding residues in the cyanopindolol-bound β_1_-AR–Gs complex, comparing with the isoproterenol-bound β_1_-AR–Gs complex.

To understand the activation of G-proteins by a GPCR bound with a partial agonist or a very weak partial agonist, we started with the structural studies. We used isoproterenol as an example of a full agonist, dobutamine as a partial agonist, and cyanopindolol as a very weak partial agonist for turkey β_1_-AR^23^ (Fig. 1a). We have solved a 2.6 Å cryo-EM structure of dobutamine-bound β_1_-AR and Gs complex (Fig. 1b, Supplementary Fig. 1, and Supplementary Table 1), and a 2.5 Å cryo-EM structure of β_1_-AR–Gs in complex with cyanopindolol (Fig. 1c, Supplementary Fig. 2, and Supplementary Table 2). We then compare and contrast these structures with the previously determined 2.6 Å cryo-EM structure of isoproterenol-bound β_1_-AR and Gs complex^20^. The well-defined density maps allowed us to build structures of β_1_-AR–Gs in the presence of an agonist, a partial agonist and a very weak partial agonist (Supplementary Fig. 3). Overall, the structures of the β_1_-AR–Gs complex in the presence of isoproterenol, dobutamine, or cyanopindolol are similar. However, there are local conformational differences, especially in the ligand-binding pockets (Fig. 2, Supplementary Figs. 4-6). Rearrangements of critical ligand-binding residues (such as Phe201^ECL2^) can be detected when comparing the isoproterenol and dobutamine-bound structures with the cyanopindolol-bound structure (Fig. 2a). While some of the interacting residues are common to all three ligands, including Trp117^3.28^, Thr118^3.29^, Asp121^3.32^, Val122^3.33^, Val125^3.36^, Phe201^ECL2^, Ser211^5.42^, Ser215^5.46^, Phe306^6.51^, Asn310^6.55^, Asn329^7.39^, and Tyr333^7.43^ (the superscript denotes the Ballestero-Weinstein numbering system)^24^, dobutamine and cyanopindolol each make a unique set of additional interactions in the orthosteric ligand-binding pocket (Fig. 2b–d, Supplementary Fig. 7). Dobutamine is additionally coordinated by the backbone carbonyl oxygen of Gly98^2.61^, and side chains of Leu101^2.64^, Val102^2.65^, Phe307^6.52^, Val326^7.36^, and Trp330^7.40^ (Fig. 2c, Supplementary Fig. 7). Cyanopindolol, on the other hand, makes additional interactions with Thr126^3.37^, Thr203^ECL2^, Ala208^5.39^, and Phe307^6.52^ on the opposing side of the orthosteric ligand-binding pocket (Fig. 2d, Supplementary Fig. 7). These shared and distinct interactions are essential for the accommodation of the three ligands with different chemical scaffolds, and are similar to those observed in the complexes of these ligands with β_1_-AR in the presence of a conformation-specific nanobody^25^ (Fig. 2, Supplementary Figs. 3, 7, 8).

**Fig. 3:**
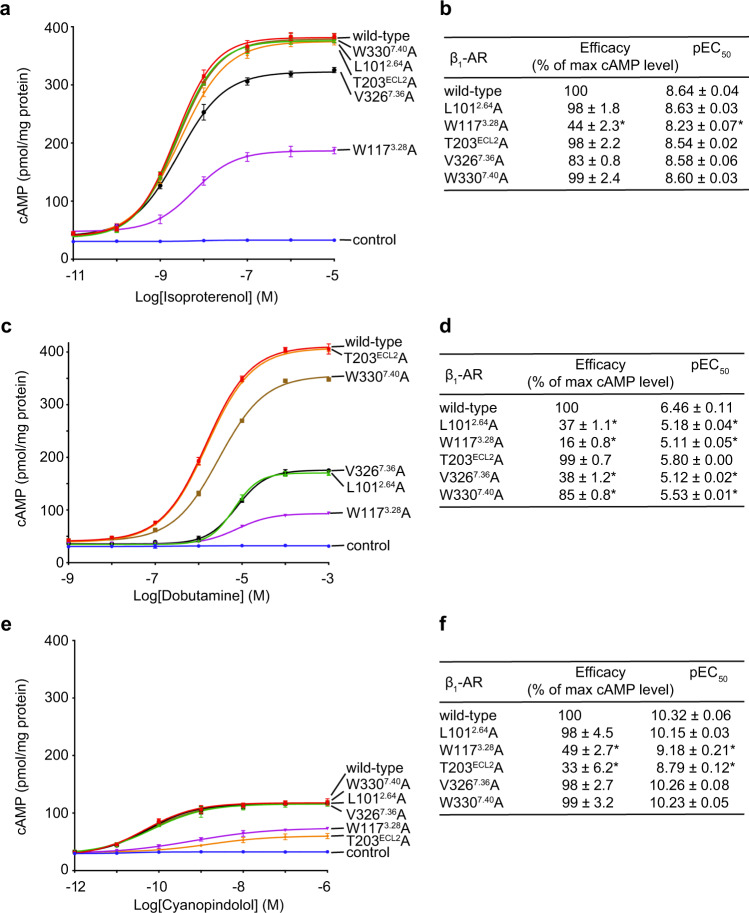
Functional studies of receptor residues involved in specific interactions with the different ligands. **a**, **b** Effects of receptor mutations on isoproterenol-induced cellular cAMP responses. **a** Dose–response data from cells expressing different β_1_-AR constructs after stimulation with isoproterenol. **b** Summary of the efficacy (the maximum cAMP level of a mutant receptor / the maximum cAMP level of the wild-type receptor) and the potency (EC_50_ values) based on the cAMP assay data shown in (**a**). **c**, **d** Effects of receptor mutations on dobutamine-induced cAMP responses. (**c**) Dose–response data from cells expressing different β_1_-ARs after stimulation with dobutamine. (**d**) Summary of the efficacy and EC_50_ values based on the cAMP assay data shown in (**c**). (**e**, **f**) Effects of mutations on cyanopindolol-induced cAMP responses. (**e**) Dose–response data from cells expressing different β_1_-ARs after stimulation with cyanopindolol. **f** Summary of the efficacy and EC_50_ values based on the cAMP assay data shown in (**e**). Data are shown as mean ± SD of three experiments. The analysis was done using the log(agonist) vs. response function of Prism 8 (GraphPad). Statistical analysis was used to compare individual mutant receptors with the wild-type receptor. *p < 0.05 (Student’s *t*-test, two-sided). Source data are provided as a Source Data file.

To functionally validate the structurally identified residues, we have mutated some shared and unique residues involved in ligand interactions (Fig. 3a–f). We mutated residues Leu101^2.64^ (for Dob), Trp117^3.28^ (for all three ligands), Thr203^ECL2^ (for Cya), Val326^7.36^ (for Dob), and Trp330^7.40^ (for Dob) to Ala. These mutants were then expressed in cells, and their responses to different concentrations of the three different ligands (isoproterenol, dobutamine, and cyanopindolol) to generate cellular cAMPs were quantified (Fig. 3a–f, Supplementary Fig. 9). While Trp117Ala mutation decreased the potency and efficacy of all three different ligands, Leu101Ala, Val326Ala, and Trp330Ala mutations only decreased the potency and efficacy of dobutamine (Fig. 3d). Thr203Ala mutation only decreased the potency and efficacy of cyanopindolol (Fig. 3f). These functional data supports the above structural studies identifying these residues involved in specific interactions with the different ligands.

### β_1_-AR conformations within the three β_1_-AR–Gs complexes

Given our focus on the activation of G-proteins by a GPCR after bound with ligands of different efficacies, we examined whether the β_1_-AR conformations in the three β_1_-AR–Gs complexes are different (Supplementary Fig. 10). At the cytoplasmic side of the receptors, GPCR activation is generally characterized by the displacement of TM5, TM6 and TM7^4^ (Supplementary Fig. 10a). Analysis of the three β_1_-AR structures shows that β_1_-ARs in the three complexes have similar overall conformations with local differences. The conformation in the intracellular half of the TM bundle is notably shifted towards that seen in the active state β_1_-AR structure^20^ (Supplementary Fig. 10a), and distinct from those of the inactive-state β_1_-AR structures^26,27^ (Supplementary Fig. 10a). In addition to these TM conformational changes, GPCR activations are characterized by the rotameric changes of several conserved motifs^28^. We compared the rotamer positions in the β_1_-ARs in these three complexes and in the inactive state β_1_-AR (Supplementary Fig. 10b–e). Residues Pro219^5.50^, Ile129^3.40^ and Phe299^6.44^ form an interface between TM5, TM3 and TM6 near the base of the ligand binding pocket in β_1_-AR and other class A GPCRs. In the active state structures of β_1_-AR, a chain of conformational rearrangements occur in these residues, in which an inward shift of Phe219^5.50^ is coupled with a rotamer switch in Ile129^3.40^, a large movement of the Phe299^6.44^ side chain, and a corresponding rotation of TM6 on the cytoplasmic side (Supplementary Fig. 10b). All three β_1_-AR structures display similar conformational changes of these residues; no intermediate conformations are observed in the presence of partial agonists (Supplementary Fig. 10b).

Another important aspect of class A GPCR activation is the rearrangement of side chains in highly conserved motifs D(E)/RY (TM3) and NPxxY (TM7), which are referred to as “micro-switches”^28^. The ionic-lock salt bridge is preserved between the side chains of Arg139^3.50^ and Asp138^3.49^ in the β_1_-AR inactive state, but it is broken in the active state structure (Supplementary Fig. 10c). Additionally, Arg139^3.50^ forms a salt bridge with Glu285^6.30^ in the inactive state of β_1_-AR^27^, but this interaction is broken in the active state of β_1_-AR (Supplementary Fig. 10c). In the active state structure, the Asp138^3.49^ side chain forms a hydrogen bond to Tyr149 in ICL2, and the Arg139^3.50^ side chain interacts with Tyr377 in the α5-helix of Gα_s_ (Supplementary Fig. 10c). The highly conserved NPxxY motif at the cytoplasmic end of TM7 is another key micro-switch of GPCR activation^28^. All three β_1_-AR structures show active state conformations of the NPxxY motif when compared to the inactive β_1_-AR (Supplementary Fig. 10d, e). Therefore, β_1_-ARs in the three different complexes, with Gs-proteins, adopt similar active state conformations, even though they are bound with ligands with different efficacies.

Each of the above three β_1_-AR–Gs complex structures represents the mean conformation of the imaged particles. Three-dimensional variability analysis (3DVA) revealed that, in all three complexes, both β_1_-AR and Gs show conformational flexibility (Supplementary Movies 1-3 for the complex of β_1_-AR–Gs with dobutamine)^29^. Supplementary Movie 1 shows the oscillating movement of β_1_-AR away or towards Gs. Supplementary Movie 2 shows the twisting of β_1_-AR along the membrane axis. Supplementary Movie 3 shows the transverse bending of β_1_-AR, as well as motions of the extracellular parts of β_1_-AR. The N-terminal coiled coil of Gβγ is very dynamic (Supplementary Movies 2 and 3). These types of motions are also seen in the complexes of β_1_-AR–Gs with isoproterenol or cyanopindolol. These analyses reveal the dynamic nature of β_1_-AR and Gs-proteins, as well as their interactions.

**Fig. 4:**
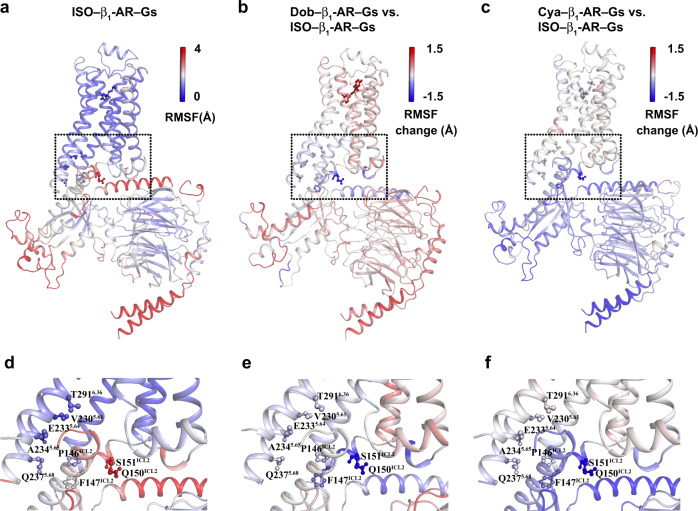
Flexibility changes of the agonist–β_1_-AR–Gs complexes observed in GaMD simulations. **a**, **d** The root-mean-square fluctuations (RMSFs) of the isoproterenol–β_1_-AR–Gs complex. **b**, **e** Changes in RMSFs of β_1_-AR and Gs when the dobutamine–β_1_-AR–Gs complex compared with the isoproterenol–β_1_-AR–Gs complex. **c**, **f** Changes in RMSFs of β_1_-AR and Gs when the cyanopindolol–β_1_-AR–Gs complex compared with the isoproterenol–β_1_-AR–Gs complex.

GaMD simulations also showed different local conformational flexibilities in the three complexes (Fig. 4, Supplementary Fig. 11). Overall, β_1_-AR underwent small fluctuations in all three complexes except for higher flexibilities in ICL1 and H8^21^ (Supplementary Fig. 11a–c). Consistent with their experimental binding affinities, cyanopindolol displayed the lowest fluctuation, while dobutamine with the highest fluctuation (Fig. 4, Supplementary Fig. 12a–c). Within the three complexes, Gs-proteins exhibited higher fluctuations than membrane-embedded β_1_-ARs (Supplementary Fig. 11a–c). The α5-helix, the α4-β5 loop, the Switch III region, and the αN-helix of Gα_s_, as well as the N-termini of Gβγ showed higher structural flexibilities (Supplementary Fig. 11a–c). Compared with the isoproterenol–β_1_-AR–Gs, the dobutamine-β_1_-AR-Gs structure showed different flexibilities in the ligand-binding pocket, TM1, ICL1, TM2, ICL2, TM4, ECL3, TM7 and H8 of β_1_-AR, as well as local regions of Gα_s_ and Gβγ (Fig. 4b). The cyanopindolol–β_1_-AR–Gs complex also showed different flexibilities in the ligand-binding pocket, TM1, ICL1, ICL2, TM5, TM6, TM7 and H8 of β_1_-AR, as well as local regions of Gα_s_ and Gβγ (Fig. 4c). Overall, the cyanopindolol–β_1_-AR–Gs complex is relatively less flexible (Fig. 4c). The residues in β_1_-AR that are involved in Gs interactions showed varied flexibilities in the three complexes (Fig. 4d–f). In addition, we simulated β_1_-AR bound by the three agonists after removing Gs from the cryo-EM structures (Supplementary Figs. 11d–f, 12d–f). In the absence of Gs, β_1_-AR displayed higher fluctuations in the ligand-binding pocket, ICL2, the cytoplasmic ends of TM5 and TM6, as well as H8, in all three complexes (Supplementary Fig. 11d–f). Furthermore, isoproterenol and dobutamine underwent higher fluctuations, while the high affinity ligand cyanopindolol remained stable (Supplementary Figures 11 d-f and 12 d-f). With the G-protein, isoproterenol became stabilized, consistent with the allosteric stabilization of agonist binding by G-proteins (Supplementary Fig. 11a–c)^30^. Overall, the GaMD simulations support our cryo-EM structural data showing local conformational differences among the three complexes.

### Cellular studies of the activation of Gs by β_1_-ARs initiated by the three different ligands

**Fig. 5:**
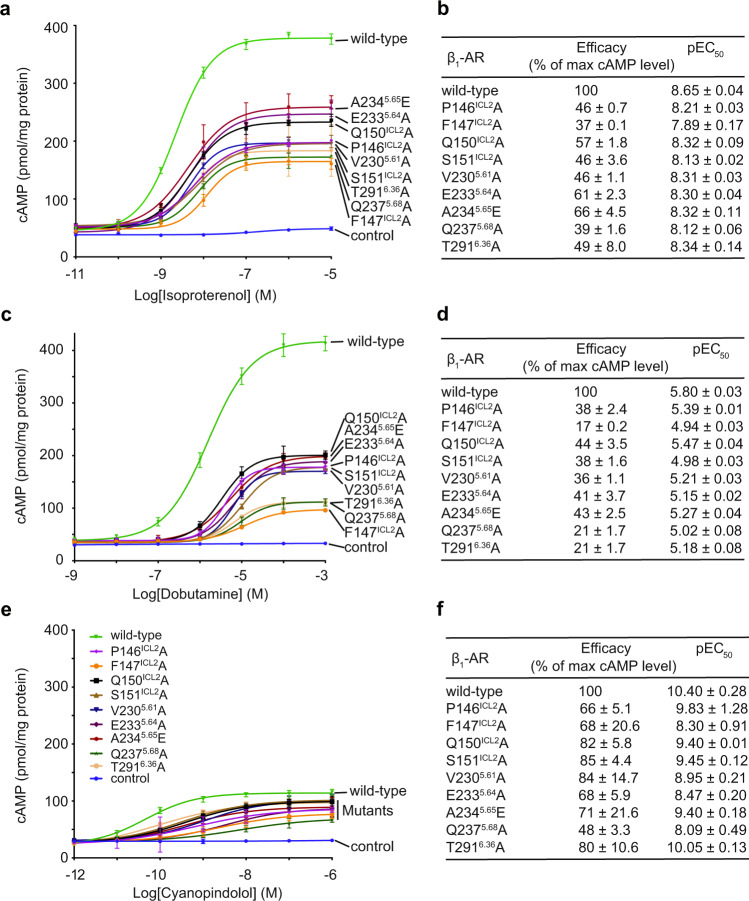
Functional studies of the signaling from β_1_-ARs initiated by a full agonist, a partial agonist, or a very weak partial agonist. **a**, **b** Effects of receptor mutations on isoproterenol-induced cAMP responses. **a** Dose–response data from cells expressing different β_1_-AR constructs after stimulation with isoproterenol. **b** Summary of the efficacy (the maximum cAMP level of a mutant receptor/the maximum cAMP level of the wild-type receptor) and EC_50_ values based on the cAMP assay data shown in (**a**). **a** and **b** are adapted from^20^ and used here for direct comparison. **c**, **d** Effects of mutations on dobutamine-induced cAMP responses. **c** Dose–response data from cells expressing different β_1_-ARs after stimulation with dobutamine. **d** Summary of the efficacy and EC_50_ values based on the cAMP assay data shown in (**c**). **e**, **f** Effects of mutations on cyanopindolol-induced cAMP responses. **e** Dose–response data from cells expressing different β_1_-ARs after stimulation with cyanopindolol. **f** Summary of the efficacy and EC_50_ values based on the cAMP assay data shown in (**e**). Data are shown as mean ± SD of three experiments. The analysis was done using the log(agonist) vs. response function of Prism 8 (GraphPad). When comparing with the wild-type receptor, all mutant receptors showed significant difference with *p* values < 0.05 (Student’s *t*-test, two-sided). The color keys for the receptor mutants are the same for (**c**) and (**e**), and are displayed in (**e**). Source data are provided as a Source Data file.

We mutated residues on β_1_-AR that participate in its interaction with Gs, and investigated whether these interacting residues contribute similarly or differently to the signaling from β_1_-AR to the cAMP response, after stimulation with the three different ligands (Fig. 5, Supplementary Fig. 9). We selected representative residues from TM5, TM6 and ICL2 since these regions contribute most to the interactions. For isoproterenol, mutations of the interacting residues in β_1_-AR reduced the magnitude of the cAMP response by 37–66% and the EC_50_ by 2 to 6-fold, confirming the importance of these interacting residues for β_1_-AR signaling to Gs^20^ (Fig. 5a, b). Similarly, the β_1_-AR mutants decreased the dobutamine-initiated cAMP response by 17–44% and the EC_50_ by 2 to 7-fold (Fig. 5c, d). Despite cyanopindolol inducing a maximum cAMP response that is only ~24% of the cAMP response induced by isoproterenol in the wild-type context, the efficacy and potency were both decreased by the β_1_-AR mutants (Fig. 5e, f). These data indicate that the interacting residues are critical for activation of Gs (and thus the signaling to the downstream cAMP response) by β_1_-AR in response to all three ligands with different efficacies. However, there are different degrees of impairments by some of these mutants (Fig. 5). For example, Gln237^5.68^Ala and Thr291^6.36^Ala had a larger effect on dobutamine-induced cAMP signaling than on isoproterenol initiated responses (Fig. 5b, d, f, Supplementary Fig. 9). Phe147^ICL2^Ala had a major effect on the cAMP responses stimulated by isoproterenol and dobutamine, than by cyanopindolol (Fig. 5b, d, f, Supplementary Fig. 9). These results indicate that there are residue-specific differences in the signaling mechanisms when bound with ligands with different efficacies.

**Fig. 6:**
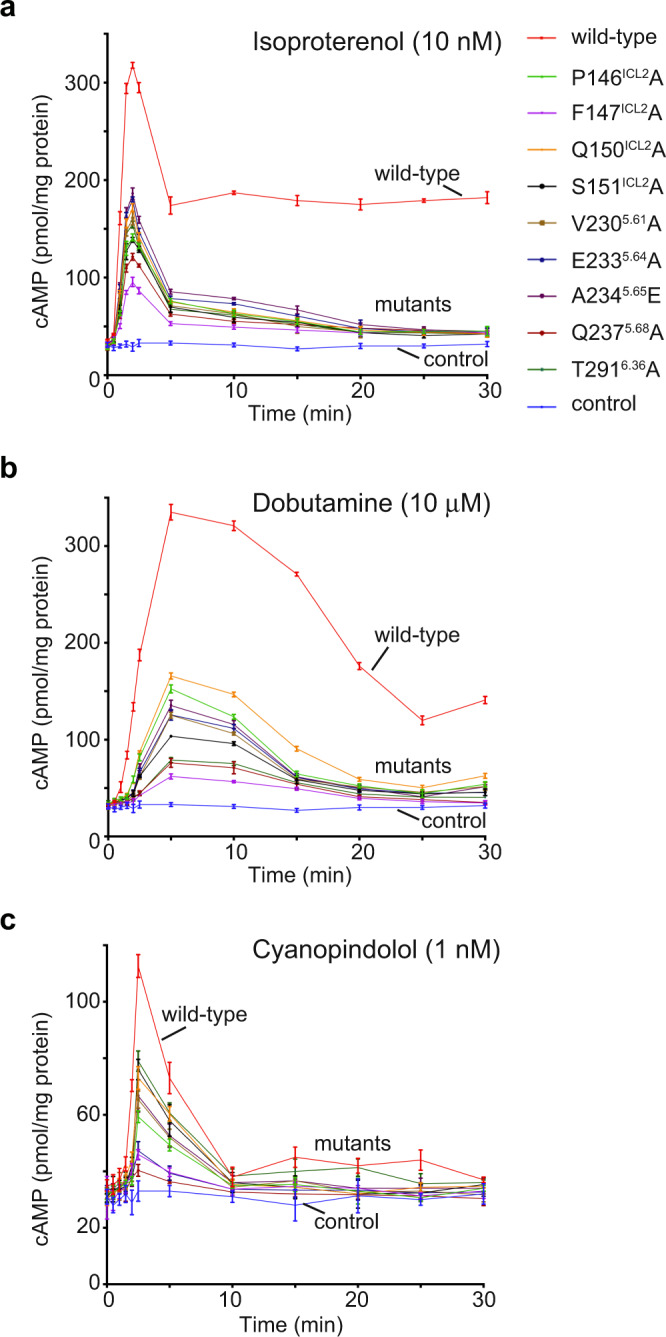
Effects of β_1_-AR mutations on the time-dependent cellular cAMP responses initiated by three different ligands. Time-dependent cAMP responses induced by isoproterenol (**a**), dobutamine (**b**), or cyanopindolol (**c**), are decreased by the β_1_-AR mutations. Data are presented as mean ± SD of three experiments. When comparing with the wild-type receptor, all mutant receptors showed significant difference with *p* values <0.05 (Student’s *t*-test, two-sided). The color keys for the receptor mutants are displayed in (**a**), and are the same for (**a**, **b**, and **c**). Source data are provided as a Source Data file.

Furthermore, in addition to the above concentration-dependent cAMP responses, we also investigated the effect of these same mutations on the kinetics of cAMP signaling through β_1_-AR (Fig. 6, Supplementary Fig. 13). We measured cAMP responses over time in the presence of near-saturating concentrations (EC_90_) of isoproterenol, dobutamine, and cyanopindolol (Fig. 6). Isoproterenol induced a quick and robust signal activation phase, and a fast signal termination phase, followed by a high sustaining plateau phase (Fig. 6a). Dobutamine generated a slower signal activation phase and an even slower signal termination phase (Fig. 6b). Cyanopindolol produced a fast activation phase and a fast termination phase without a sustaining phase (Fig. 6c). Mutations of the interacting residues decreased all phases of the signaling responses (Fig. 6, Supplementary Fig. 13). We noticed that there are ligand-specific differences by some of these mutants affecting the rates of activation or termination (Supplementary Fig. 13). For example, Gln237^5.68^Ala had a larger effect on the rate of activation by cyanopindolol than by isoproterenol (Supplementary Fig. 13). Pro146^ICL2^Ala and Phe147^ICL2^Ala had similar effects on the rates of termination by isoproterenol and cyanopindolol, but they had different effects on the rates of termination by dobutamine (Supplementary Fig. 13). We should note that the desensitization phase also depends on the receptor interaction with other proteins (such as G-protein-coupled receptor kinases and arrestins) in cells. These data affirm that various aspects of the downstream signaling induced by ligands with different efficacies are affected by impairing the β_1_-AR and Gs interactions.

### Biochemical studies of the stability of the intermediate state complexes of β_1_-AR and Gs when bound with different ligands

**Fig. 7:**
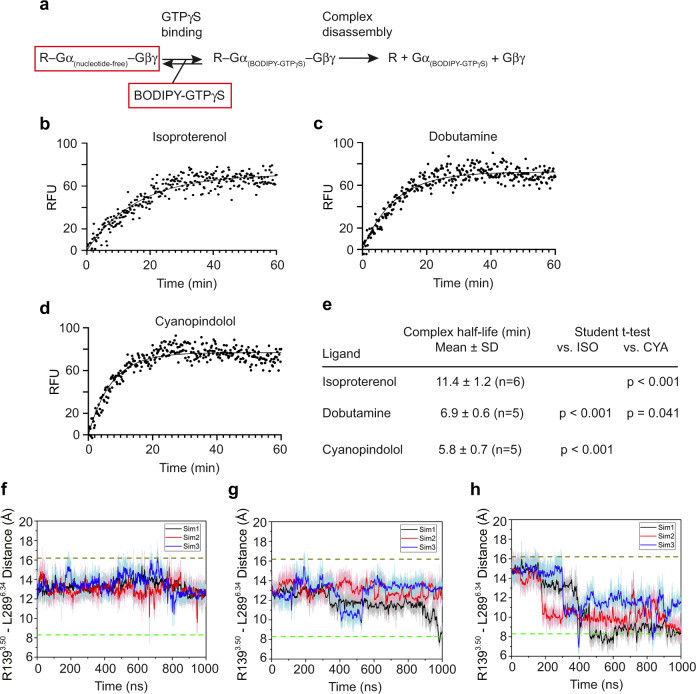
Biochemical studies and GaMD simulations of the stability of the complexes of β_1_-AR and Gs when bound with a full agonist, a partial agonist, or a very weak partial agonist. **a** A schematic diagram represents the chemical process from BODIPY-GTPγS binding to the complex of ligand–receptor–Gα(nucleotide-free)–Gβγ), leading to the formation of the transient R–G(BODIPY-GTPγS) bound complex and the subsequent complex disassembly. **b**–**e** BODIPY-GTPγS binding to the transition state complex in the presence of isoproterenol (**b**), dobutamine (**c**), or cyanopindolol (**d**). Units reported as relative fluorescent units (RFU). One representative experiment from five or six independent experiments with similar results is shown for each case. **e** Summary of the half-life values. Data are shown as mean ± SD of five or six independent experiments. Two-sided *P* values are from Student’s *t*-test. **f**–**h** Ligand-dependent structural dynamics of β_1_-AR in the absence of Gs. The distance between the cytoplasmic ends of TM3 and TM6 (measured as the distance between the Cα atoms of Arg139^3.50^ and Leu289^6.34^) is calculated over the indicated time. Three GaMD simulations (black, red, blue) were performed. Lines depict the running average over 2 ns. The top dash lines indicate the distance observed in the cryo-EM structures, and the bottom dash lines indicate the distance observed in the inactive β_1_-AR (PDB 4GPO).

Finally we used biochemical studies to investigate the activation of Gs by β_1_-ARs when bound with ligands of different efficacies. GPCRs are enzymes that catalyze the guanine-nucleotide exchange on G-proteins^31^. The nucleotide-free state resolved in the structures with isoproterenol, dobutamine, and cyanopindolol represents an intermediate state in the guanine-nucleotide exchange reaction coordinate^32^ (Fig. 7a), and thus we hypothesized that differences in the free energy, and thus stability, of the complex would have profound effects on the activation of G-proteins^33,34^. To investigate whether ligands with different efficacies have different effects on the stability of the intermediate state, we prepared the complex of ligand-bound β_1_-AR-Gs (nucleotide-free), and quantified the stability of the intermediate state complex (Fig. 7a). Fluorescently labeled BODIPY-GTPγS binding to the ligand-bound β_1_-AR–Gs (nucleotide-free) complex eventually leads to the dissociation of the complex to produce the fluorescently labeled product Gα_s_(BODIPY-GTPγS), which is detected by an increase in fluorescence (the β_1_-AR–Gα_s_(BODIPY-GTPγS)–Gβγ intermediate is very transient)^31,35^ (Fig. 7a–d). The data were then fitted by nonlinear association analyses and half-life values (*t*_1/2_ = ln 2/*k*) were calculated based on the rate constant (Fig. 7b–e). The sustained fluorescence levels during the assay time period reflect the known slow dissociation rate of BODIPY-GTPγS from the free Gα subunit^36–38^. The isoproterenol–β_1_-AR–Gs complex has the longest half-life and is thus the most stable among the three complexes (Fig. 7e). The cyanopindolol–β_1_-AR–Gs complex is the least stable, displaying the shortest half-life (Fig. 7e). The dobutamine–β_1_-AR–Gs complex displays an intermediate stability (Fig. 7e).

The stability of ligand-bound β_1_-ARs in a G-protein compatible conformation (the residence time of β_1_-AR in the activate state) is further investigated by GaMD simulations (Fig. 7f–h). As mentioned before, the main conformational change of β_1_-AR during its activation is the outward movement of the cytoplasmic end of TM6 (Supplementary Fig. 10). Thus the distance between the cytoplasmic ends of TM3 and TM6 (measured by the distance between Arg139^3.50^ and Leu289^6.34^) can be used as a measurement of the activation status of β_1_-AR^39^. Removal of G_s_ from the ligand–β_1_-AR–Gs complex leads to the deactivation of β_1_-AR; this is reflected by the decreased TM3-TM6 distance in GaMD simulations of the cyanopindolol–β_1_-AR complex (Fig. 7h, Supplementary Fig. 14i) and the dobutamine–β_1_-AR complex (Fig. 7g, Supplementary Fig. 14h). β_1_-ARs in these two complexes were more dynamic than in the isoproterenol-bound form (Fig. 7f–h, Supplementary Fig. 14g–i). With isoproterenol, β_1_-AR mostly adopted a state with a TM3-TM6 distance of ~12–14 Å (Fig. 7f, Supplementary Fig. 14g). When bound with dobutamine, β_1_-AR transitioned to the inactive state with a TM3-TM6 distance of ~8.3 Å in one of the three GaMD simulations (Fig. 7g, Supplementary Fig. 14h). Cyanopindolol-bound β_1_-AR transitioned to the inactive state within ~400–700 ns in all three GaMD simulations (Fig. 7h, Supplementary Fig. 14i). These GaMD simulations thus reveal that ligands with higher efficacies are able to maintain β_1_-AR in the active state for longer time. These data are consistent with our above biochemical data.

## Discussion

Our data shows that the structures of the same GPCR–G-protein complexes bound with ligands of different efficacies have overall similar configurations with local conformational differences. From our cryo-EM structures, β_1_-ARs in the three complexes are all in the fully active state. We did not observe a partial activation process in the critical activation microswitch residues in the complexes with a partial agonist or a very weak partial agonist. There are local conformational differences, for example, in the ligand-binding pocket, TM1, ICL1, TM2, ICL2, TM4, ECL3, TM7 and H8 of β_1_-AR. Residue specific differences were confirmed by functional studies using mutant β_1_-ARs in the ligand-binding pockets and in Gs-interaction regions. In the absence of G-proteins, β_1_-ARs bound with ligands with different efficacies were in the inactive state^40^. As shown here, in the presence of G-proteins, β_1_-ARs bound with ligands of different efficacies were in the active state. The conformational changes from the inactive to the active state of β_1_-ARs for all three ligands are similar to other Class A GPCRs (Supplementary Fig. 10). When bound to β_1_-ARs in the presence of Gs or a conformation-specific nanobody, agonists bind tighter with lower RMSDs when compared with the structures without G-proteins^25^ (Supplementary Fig. 12). While our manuscript under review, cryo-EM structures of several GPCR–G-protein complexes in the presence of partial agonists were solved, and the overall structures were similar to the full agonist-bound complexes, with some local conformational differences^41–43^. These are consistent with our observations here. Our cellular functional studies show that the residues on β_1_-ARs that interact with Gs are critical for the activation of Gs and the downstream cAMP response since mutations of these residues decreased the efficacy and potency of the cAMP response initiated by isoproterenol, dobutamine and cyanopindolol. We should note that there are some residue-specific differences in their effects on cAMP response initiated by different ligands. These might reflect the local structural differences and the different stabilities of the three complexes.

Our data suggests that the efficacy of the ligand-bound GPCR in catalyzing G-protein activation is also correlated with the stability of the intermediate state of the ligand–GPCR–G-protein complex, which is a complement to the conformation selection model. Our biochemical studies and GaMD simulations show that a full agonist-bound GPCR–G-protein intermediate state complex is more stable than a partial agonist-bound GPCR–G-protein intermediate state complex. Our observation on the stability of the entire ligand–GPCR–G-protein complex is consistent with previous fluorescence spectroscopy experiments showing that a full agonist stabilized the binary complex of β_2_-AR–Gs(nucleotide free) better than a partial agonist^44^. Recently, it has been shown that positive allosteric modulators increase the agonist and receptor (adenosine A_1_ receptor) efficacy by stabilizing the ligand–GPCR–G protein complex^39^. Future investigations should integrate the thermodynamic and kinetic reaction controls of G-protein activations by GPCRs.

## Methods

### Expression and purification of β_1_-AR, Gα_s_, Gβ_1_, Gγ_2_ and Nb35

β_1_-AR protein was purified as described previously^20,27^. The turkey β_1_-AR construct β_1_-AR(H12) used in this study was similar to the functional β_1_-AR(H0) construct described previously with some modifications^20,27^. A signal peptide, FLAG tag, PreScission protease cleavage site and T4 lysozyme were fused to the N- terminus with a double-alanine linker, and another PreScission protease cleavage site and His_6_ tag were added to the C-terminus. β_1_-AR was expressed and purified from Sf9 insect cells grown in ESF 921 protein-free medium (Expression Systems)^20^. Cells were grown to 2–3 million cells per ml before 100 ml of baculoviruses were added for infection. 48 hrs later, cells were harvested by centrifugation, flash frozen in liquid nitrogen and stored at −80 °C until use. For membrane preparation, cell pellets were lysed by sonication in a buffer containing 20 mM Tris, pH 8, 1 mM EDTA and protease inhibitor cocktail (Sigma) and washed once more using the same buffer. Purified membranes were resuspended in 20 mM Tris, pH 8, 0.2 mM EDTA, and protease inhibitor cocktail and flash frozen in liquid nitrogen and stored at −80 °C. For protein purification, membrane preparations were first thawed in 20 mM Tris, pH 8, 350 mM NaCl, and protease inhibitor cocktail. 1 mM isoproterenol (Sigma) was then added and the mixture was stirred for 1 h at 4 °C and the membranes were then solubilized in 20 mM Tris, pH 8, 350 mM NaCl, 1% n-Dodecyl-β-D-Maltopyranoside (DDM, Anatrace), 1 mM isoproterenol and protease inhibitor cocktail for 1 hr at 4 °C. The DDM concentration was then reduced to 0.5% by adding equal volume of 20 mM Tris, pH 8, 350 mM NaCl, and 1 mM isoproterenol and the mixture was stirred for another 1 hr at 4 °C. The preparation was clarified by ultracentrifugation at 142,000 g for 30 min at 8 °C. The supernatant was then incubated with Ni-NTA resin (Qiagen) with stirring at 4 °C with 8 mM imidazole. After 4 hrs, the resin was collected by centrifugation and washed three times with 20 mM Tris, pH 8, 500 mM NaCl, 0.05% DDM, 1 mM isoproterenol, and 20 mM imidazole and one time with 20 mM Tris, pH 8, 150 mM NaCl, 0.05% DDM, 1 mM isoproterenol, and 20 mM imidazole. β_1_-AR was then eluted from the resin with 20 mM Tris, pH 8, 150 mM NaCl, 0.05% DDM, 1 mM isoproterenol, and 200 mM imidazole. The elution was concentrated and further purified by size-exclusion chromatography using a Superdex 200 Increase 10/300 column (GE Healthcare) pre-equilibrated with 20 mM Tris, pH 8, 150 mM NaCl, 0.02% Lauryl Maltose Neopentyl Glycol (LMNG, Anatrace), 1 mM isoproterenol. Dobutamine- and cyanopindolol-bound β_1_-AR proteins were purified using the same protocol with 200 µM dobutamine and 50 µM cyanopindolol present during purification. Purified β_1_-AR was concentrated to 4 mg/ml and either used immediately for complex assembly or flash frozen in liquid nitrogen and stored at −80 °C.

The recombinant wild-type bovine Gα_s_ was purified from *E. coli* strain BL21(DE3)^20,45^. This Gα_s_ construct had an N-terminal GST tag that was removable through a PreScission protease cleavage site. Cells were grown in 2 × YT medium at 37 °C until OD_600_ reached 0.6. Protein expression was then induced by 75 µM IPTG and continued for 16 h at 16 °C. Cells were harvested by centrifugation, flash frozen in liquid nitrogen and stored at −80 °C. For protein purification, cell pellets were thawed in a lysis buffer containing 20 mM HEPES, pH 7, 150 mM NaCl, 10% glycerol, 5 mM β-mercaptoethanol, 2 mM MgCl_2_, 1 mM EDTA, 10 µM GDP, 0.1 mg/ml lysozyme, 0.2 mM PMSF and protease inhibitor cocktail, and further lysed by sonication. Cell debris was removed by centrifugation at 20,000 g for 40 min 4 °C. Supernatant was then collected and incubated with Glutathione resin (Pierce) with stirring for 1 hr at 4 °C. Resin was then washed four times with 20 mM HEPES, pH 7, 150 mM NaCl, 10% glycerol, 5 mM β-mercaptoethanol, 2 mM MgCl_2_, 1 mM EDTA, and 10 µM GDP. To remove the GST tag, PreScission protease was added to the beads at 1:10 (w:w) protease: GST-Gα_s_ ratio and the mixture was rocked overnight at 4 °C with 2 mM DTT. Untagged Gα_s_ was concentrated and further purified by size-exclusion chromatography using a Superdex 200 Increase 10/300 column pre-equilibrated with 20 mM HEPES, pH 7, 150 mM NaCl, 10% glycerol, 5 mM β-mercaptoethanol, 1 mM MgCl_2_, 1 mM EDTA, 20 µM GDP. Purified Gα_s_ was concentrated to 6 mg/ml, flash frozen in liquid nitrogen and stored at −80 °C.

Recombinant bovine Gβ_1_ and bovine His_6_-tagged soluble Gγ_2_(C68S) were co-expressed and purified from Sf9 insect cells^20^. 25 ml of each baculovirus were co-infected into Sf9 cells when the insect cell culture reached a cell density at 3 million cells per ml. 48 h post infection, cells were harvested by centrifugation, flash frozen in liquid nitrogen and stored at −80 °C. Cell pellets were thawed in 25 mM HEPES pH 7, 150 mM NaCl, 2 mM β-mercaptoethanol, and protease inhibitor cocktail. Cells were lysed by sonication and cell debris were removed by centrifugation at 142,000 g for 30 min. Supernatant was collected and incubated with Ni-NTA resin with stirring for 1.5 h at 4 °C. Resin was then washed three times with 25 mM HEPES pH 7, 150 mM NaCl, 2 mM β-mercaptoethanol, and 25 mM imidazole, and Gβ_1_γ_2_ was eluted as a complex with 25 mM HEPES pH 7, 150 mM NaCl, 2 mM β-mercaptoethanol, and 250 mM imidazole. Eluted protein was concentrated and further purified using a Superdex 200 Increase 10/300 column pre-equilibrated with 25 mM HEPES pH 7, 150 mM NaCl, and 2 mM β-mercaptoethanol. Purified Gβ_1_γ_2_ protein was concentrated to 8 mg/ml, flash frozen in liquid nitrogen and stored at −80 °C.

Nb35-His_6_ was expressed in the periplasm of *E. coli* strain BL21(DE3)^20^. Cells were grown in LB medium at 37 °C until OD_600_ reached 0.6. Protein expression was then induced by 75 µM IPTG and Nb35 was further expressed for 18 h at 16 °C. Cells were then harvested, flash frozen in liquid nitrogen and stored at −80 °C. For protein purification, cells were lysed by sonication in a lysis buffer containing 20 mM HEPES pH 7, 100 mM NaCl, 5 mM MgCl_2_, 0.1 mM lysozyme, and protease inhibitor cocktail. After removal of the cell debris by centrifugation at 20,000 g for 30 min, supernatant was collected and incubated with Ni-NTA resin with stirring for 1.5 hrs at 4 °C. Resin was then washed three times with 20 mM HEPES pH 7, 100 mM NaCl, and 25 mM imidazole. Nb35 was eluted with 20 mM HEPES pH 7, 100 mM NaCl, and 250 mM imidazole. Eluted Nb35 protein was dialyzed against 1 L of 20 mM HEPES pH 7, 100 mM NaCl overnight at 4 °C. Dialyzed protein was concentrated to 3 mg/ml, flash frozen in liquid nitrogen and stored in −80 °C.

### Protein complex assembly and purification

To assemble the β_1_-AR-Gs-Nb35 complex with different ligands bound, Gα_s_, Gβ_1_γ_2_ and Nb35 were mixed at 1:1:1.5 molar ratios in the presence of 2 mM MgCl_2_. The mixture was incubated for 30 min at room temperature and then mixed with β_1_-AR at 1.2:1 ratio in the presence of isoproterenol, dobutamine or cyanopindolol. The mixture was diluted with buffer containing 10 mM HEPES pH 7, 100 mM NaCl, 0.1 mM TCEP, 0.02% LMNG, and 2 mM MgCl_2_ to bring the volume to 500 µl. The final concentration of three different ligands in the mixture was 1 mM, 200 µM and 50 µM of isoproterenol, dobutamine and cyanopindolol, respectively. This mixture was incubated for another 30 min at room temperature before 0.4 U Apyrase (Sigma) was added. After additional 30 min room temperature incubation with Apyrase, the mixture was centrifuged at 16,000 g for 10 min to remove any precipitants. The supernatant was then loaded onto a Superdex 200 Increase 10/300 column pre-equilibrated with 10 mM HEPES pH 7, 100 mM NaCl, 0.1 mM TCEP, 0.02% LMNG and 40 uM corresponding ligands. The elution fractions from a single peak containing pure β_1_-AR-Gs-Nb35 complex was concentrated to ~1.8 mg/ml and used directly for making cryo-EM grids.

### Cryo-EM data collection

Four microlitre of protein complex was applied to a glow-discharged 400 mesh gold Quantifoil R1.2/1.3 holey carbon grids (Quantifoil Micro Tools), and subsequently vitrified using Vitrobot Mark IV (Thermo Fisher Scientific/FEI). Images were collected at liquid nitrogen temperature on a Titan Krios electron microscope (Thermo Fisher Scientific/FEI) operated at 300 kV accelerating voltage, at a nominal magnification of ×22,500 using a Gatan K3 direct electron detector (Gatan, Inc.) with SerialEM3.7. For cyanopindolol, a total of 10,000 micrographs were collected between −1.0 and −2.3 μm defocus. For dobutamine, a total of 9305 micrographs were collected between −1.0 and −2.3 μm. The improved DQE of the K3 enabled data acquisition at lower accumulated doses, with a final dose of 28 e^−^/Å^2^. The dose rate of 20 e^−^/pix/s was fractionated over 1.5 s into 60 frames.

### Image processing, 3D reconstructions, modeling and refinement

Full-frame motion correction was performed in Relion 3.1 using MotionCor2^46^. CTF estimation was performed in Relion 3.1 using CTFFind4^47^. Relion 3.1^48^ Laplacian-of-Gaussian picking with minimum and maximum dimensions of 76 Å and 119 Å was used to heavily over-pick at a rate of approximately 2300 particles per micrograph. The resulting particle stacks of 18 million (cyanopindolol) and 17 million (dobutamine) particles was Fourier-cropped and processed through multiple rounds of heterogeneous classification in CryoSparc v2.14.2^49^, steadily decreasing the cropping factor as junk was removed and resolution improved (Supplementary Figs. 1 and 2). 2D classification confirmed that the majority of particles were false positives, receptor alone or G-proteins alone. The final stacks of intact complexes were 2.9 million (cyanopindolol) and 2.6 million (dobutamine) particles. Further classification converged on final high-resolution stacks of 657,613 (cyanopindolol) and 440,739 (dobutamine) particles that were then subjected to Local CTF Refinement procedures in CryoSparc v2.14.2 followed by Bayesian Polishing in Relion 3.1, and finally Global CTF Refinement in CryoSparc v2.14.2 to improve higher order aberrations (Supplementary Figs. 1 and 2). Final high-resolution reconstructions were subjected to Local Refinement with Non-Uniform Refinement in CryoSparc v2.14.2 for β_1_-AR and G-proteins independently. The Local Refinement maps showed significantly improved features over the consensus maps, both with resolutions better than 2.5 Å (cyanopindolol) and 2.7 Å (dobutamine) Supplementary Figs. 1 and 2). All maps underwent the density modification (Resolve CryoEM) procedure in Phenix dev-3765, further improving the resolution^50^ (Supplementary Figs. 1 and 2). The resulting maps were super-sampled in Coot v0.9-pre^51^ to 0.71 Å per pixel with a 384-voxel box to bring out features at high resolution. The initial models of β_1_-AR, Gα_s_, Gβ_1_γ_2_, and Nb35 were derived from the cryo-EM structure of the complex of isoproterenol–β_1_-AR–Gs (PDB ID: 7JJO). Concurrently with the data processing, the models were built in Coot v0.9-pre and Real-Space Refined in Phenix dev-3765^52^ as resolutions improved, enabling a final composite map to be derived from the model and the two super-sampled local refinement maps using the Combine Focused Maps feature in Phenix dev-3765. Final rounds of Phenix dev-3765 Real-Space Refinement against the final composite map yielded the final published models for cyanopindolol and dobutamine.

### Gaussian accelerated molecular dynamics (GaMD)

GaMD is an enhanced sampling method that works by adding a harmonic boost potential to reduce the system energy barriers^21,22^. When the system potential $V\left(\mathop{r}\limits^{ \rightharpoonup }\right)$ is lower than a reference energy E, the modified potential ${V}^{* }\left(\mathop{r}\limits^{ \rightharpoonup }\right)$ of the system is calculated as:

$${V}^{* }\left(\mathop{r}\limits^{ \rightharpoonup }\right)=V\left(\mathop{r}\limits^{ \rightharpoonup }\right)+\triangle V\left(\mathop{r}\limits^{ \rightharpoonup }\right)$$

1 $$\triangle V\left(\mathop{r}\limits^{ \rightharpoonup }\right)=\left\{\begin{array}{cc}\frac{1}{2}k{\left(E-V\left(\mathop{r}\limits^{ \rightharpoonup }\right)\right)}^{2},&V\left(\mathop{r}\limits^{ \rightharpoonup }\right) < E\\ 0, \hfill & \;\; V\left(\mathop{r}\limits^{ \rightharpoonup }\right)\ge E,\end{array}\right.$$

where *k* is the harmonic force constant. The two adjustable parameters E and k are automatically determined on three enhanced sampling principles. First, for any two arbitrary potential values ${v}_{1}\left(\mathop{r}\limits^{ \rightharpoonup }\right)$ and ${v}_{2}\left(\mathop{r}\limits^{ \rightharpoonup }\right)$ found on the original energy surface, if ${V}_{1}\left(\mathop{r}\limits^{ \rightharpoonup }\right) < {V}_{2}\left(\mathop{r}\limits^{ \rightharpoonup }\right)$, $\triangle V$ should be a monotonic function that does not change the relative order of the biased potential values; i.e., ${V}_{1}^{* }\left(\mathop{r}\limits^{ \rightharpoonup }\right) < {V}_{2}^{* }\left(\mathop{r}\limits^{ \rightharpoonup }\right)$. Second, if ${V}_{1}\left(\mathop{r}\limits^{ \rightharpoonup }\right) < {V}_{2}\left(\mathop{r}\limits^{ \rightharpoonup }\right)$, the potential difference observed on the smoothened energy surface should be smaller than that of the original; i.e., ${V}_{2}^{* }\left(\mathop{r}\limits^{ \rightharpoonup }\right){-V}_{1}^{* }\left(\mathop{r}\limits^{ \rightharpoonup }\right) < {V}_{2}\left(\mathop{r}\limits^{ \rightharpoonup }\right){-V}_{1}\left(\mathop{r}\limits^{ \rightharpoonup }\right)$. By combining the first two criteria and plugging in the formula of ${V}^{* }\left(\mathop{r}\limits^{ \rightharpoonup }\right)$ and $\triangle V$, we obtain

2 $${V}_{{\max }}\le E\le {V}_{m{in}}+\frac{1}{k},$$

Where ${V}_{{\min }}$ and ${V}_{{\max }}$ are the system minimum and maximum potential energies. To ensure that Eq. 2 is valid, *k* has to satisfy: $k\le 1/\left({V}_{{\max }}-{V}_{{\min }}\right)$. Let us define: $k={k}_{0}\cdot 1/\left({V}_{{\max }}-{V}_{{\min }}\right)$, then $0{ < k}_{0}\le 1$. Third, the standard deviation (SD) of $\triangle V$ needs to be small enough (i.e. narrow distribution) to ensure accurate reweighting using cumulant expansion to the second order: ${\sigma }_{\triangle V}=k\left(E-{V}_{{avg}}\right){\sigma }_{V}\le {\sigma }_{0}$, where ${V}_{{avg}}$ and ${\sigma }_{V}$ are the average and SD of $\triangle V$with ${\sigma }_{0}$ as a user-specified upper limit (e.g., ${10k}_{B}T$) for accurate reweighting. When E is set to the lower bound $E={V}_{{\max }}$ according to Eq. 2, ${k}_{0}$ can be calculated as

3 $${k}_{0}={{\min }}\left(1.0,{k}_{0}^{{\prime} }\right)={\min }\left(1.0,\frac{{\sigma }_{0}}{{\sigma }_{V}}\cdot \frac{{V}_{{\max }}-{V}_{{\min }}}{{V}_{{\max }}-{V}_{{avg}}}\right),$$

Alternatively, when the threshold energy E is set to its upper bound $E={V}_{{\min }}+1/k$, ${k}_{0}$is set to:

4 $${k}_{0}={k}_{0}^{{\prime} {\prime} }\equiv \left(1-\frac{{\sigma }_{0}}{{\sigma }_{V}}\right) \cdot \frac{{V}_{{\max }}-{V}_{{\min }}}{{V}_{{avg}}-{V}_{{\min }}},$$

If ${k}_{0}^{{\prime} {\prime} }$is calculated between 0 and 1. Otherwise, ${k}_{0}$is calculated using Eq. 3.

### System setup

The isoproterenol–β_1_-AR–G_s_, dobutamine–β_1_-AR–G_s_ and cyanopindolol–β_1_-AR–G_s_ cryo-EM structures were used for setting up simulation systems. The initial structures of isoproterenol–β_1_-AR, dobutamine–β_1_-AR and cyanopindolol–β_1_-AR were obtained by removing G_s_ from the isoproterenol–β_1_-AR–G_s_, dobutamine–β_1_-AR–G_s_ and cyanopindolol–β_1_-AR–G_s_ cryo-EM structures. According to previous findings, ICL 3 is highly flexible and removal of ICL3 does not appear to affect GPCR function^53,54^. ICL3 missing in the cryo-EM structures was thus omitted in the GaMD simulations. Similarly^55^, the α-helical domain of G_s_ missing in the cryo-EM structures was not included in the simulation models. This was based on earlier simulation of the β_2_-AR–G_s_ complex, which showed that the α-helical domain fluctuated substantially^53^. Other missing residues in G_s_ were modelled using SWISS Modeller^56^. All chain termini were capped with neutral groups (acetyl and methylamide). All the disulphide bonds in the complexes that were resolved in the cryo-EM structures were maintained in the simulations. Using the *psfgen* plugin in VMD^57^, missing atoms in protein residues were added and all protein residues were set to the standard CHARMM protonation states at neutral pH. For each of the complex systems, the receptor was inserted into a palmitoyl-oleoyl-phosphatidyl-choline (POPC) bilayer with all overlapping lipid molecules removed using the membrane plugin in VMD^57^. The system charges were then neutralized at 0.15 M NaCl using the solvate plugin in VMD^57^. The simulation systems were summarized in Supplementary Table 3.

### Simulation protocol

The CHARMM36m parameter set^58–60^ was used for the proteins and lipids. Force field parameters of the agonists (isoproterenol, dobutamine and cyanopindolol) were obtained from the ParamChem web server^61^. Force field parameters with high penalty were optimized used with FFParm^62^. GaMD simulations of these systems followed a similar protocol used in previous studies of GPCRs^55,63,64^. For each of the complex systems, initial energy minimization, thermalization, and 20 ns cMD equilibration were performed using NAMD2.12^65^. A cutoff distance of 12 Å was used for the van der Waals and short-range electrostatic interactions and the long-range electrostatic interactions were computed with the particle-mesh Ewald summation method^66^. A 2-fs integration time step was used for all MD simulations and a multiple-time-stepping algorithm was used with bonded and short-range non-bonded interactions computed every time step and long-range electrostatic interactions every two timesteps. The SHAKE algorithm^67^ was applied to all hydrogen-containing bonds. The NAMD simulation started with equilibration of the lipid tails. With all other atoms fixed, the lipid tails were energy minimized for 1,000 steps using the conjugate gradient algorithm and melted with a constant number, volume, and temperature (NVT) run for 0.5 ns at 310 K. The four systems were further equilibrated using a constant number, pressure, and temperature (NPT) run at 1 atm and 310 K for 10 ns with 5 kcal/(mol^.^ Å^2^) harmonic position restraints applied to the protein and ligand atoms. Final equilibration of each system was performed using an NPT run at 1 atm pressure and 310 K for 0.5 ns with all atoms unrestrained. After energy minimization and system equilibration, conventional MD simulations were performed on each system for 20 ns at 1 atm pressure and 310 K with a constant ratio constraint applied on the lipid bilayer in the X-Y plane.

With the NAMD output structures, the system topology and CHARMM36m force field files, the *ParmEd* tool in the AMBER package^68^ was used to convert the simulation files into the AMBER format. The GaMD module implemented in the GPU version of AMBER20^21,68^ was then applied to perform the simulations. GaMD simulations of the isoproterenol–β_1_-AR–G_s_, dobutamine–β_1_-AR–G_s_ and cyanopindolol–β_1_-AR–G_s_ included an 8.5-ns short cMD simulation used to collect the potential statistics for calculating GaMD acceleration parameters, a 68-ns equilibration after adding the boost potential, and finally three independent 500-ns GaMD production simulations with randomized initial atomic velocities. The average and SD of the system potential energies were calculated every 850,000 steps (1.7 ns). GaMD simulations of isoproterenol–β_1_-AR, dobutamine–β_1_-AR and cyanopindolol–β_1_-AR with smaller system sizes included a 2.8-ns short cMD simulation used to collect the potential statistics for calculating GaMD acceleration parameters, a 50.4-ns equilibration after adding the boost potential, and finally three independent 1000-ns GaMD production simulations with randomized initial atomic velocities. The average and SD of the system potential energies were calculated every 280,000 steps (0.56 ns). All GaMD simulations were run at the “dual-boost” level by setting the reference energy to the lower bound. One boost potential was applied to the dihedral energetic term and the other to the total potential energetic term. The upper limit of the boost potential SD, σ_0_ was set to 6.0 kcal/mol for both the dihedral and the total potential energetic terms. Similar temperature and pressure parameters were used as in the NAMD simulations.

### Simulation analysis

CPPTRAJ^69^ and VMD^57^ were used to analyze the GaMD simulations. The root-mean square deviations (RMSDs) of the agonists (isoproterenol, dobutamine and cyanopindolol) relative to the cryo-EM structures and the distance between the receptor TM3 and TM6 intracellular ends (measured by the distance between the Cα atoms of receptor residues Arg139^3.50^ and Leu289^6.34^) were selected as reaction coordinates. Time courses of these reaction coordinates obtained from the GaMD simulation were plotted in Fig. 7f–h, Supplementary Figs. 12, 14a–c. Root-mean-square fluctuations (RMSFs) were calculated for the protein residues and agonists, averaged over three independent GaMD simulations and color-coded for schematic representation of each complex system (Fig. 4, Supplementary Fig. 11). The PyReweighting^70^ toolkit was applied to reweight GaMD simulations to recover the original free energy profiles of the simulation systems. 2D free energy profiles were computed using the combined trajectories from all the three independent GaMD simulations for each system with agonist RMSD and TM3-TM6 distance as reaction coordinates (Supplementary Figs. 14d–i). A bin size of 1.0 Å was used for agonist RMSD and TM3-TM6 distance. The cutoff was set to 500 frames for 2D free energy calculations.

### cAMP assay

CHO cells (transfected with a control empty vector or wild-type or mutant turkey β_1_-AR) were plated onto six-well plates, and were pre-incubated with culture medium buffered with 0.5 mM IBMX for 30 min at 37 °C^20^. After washing twice with HEM buffer (20 mM HEPES, pH 7.4, 135 mM NaCl, 4.7 mM KCl, 1.2 mM MgSO_4_, 2.5 mM NaHCO_3_, 0.1 mM Ro-20-1724, 0.5 U/ml adenosine deaminase, and 1 mM IBMX), cells were treated with different concentrations of ligands in HEM buffer for 5 min for the dose–response studies. For the time course studies, cells were stimulated with ligands (10 nM for isoproterenol, 10 μM for dobutamine, and 1 nM for cyanopindolol) for 0, 0.5, 1, 1.5, 2, 2.5, 5, 10, 15, 20, 25, 30 min at 37 °C. After culture medium removal, cells were treated with 0.1 M HCl for 10 minutes at room temperature. After centrifugation, the supernatant was used for cAMP quantification using the Direct Cyclic AMP Enzyme Immunoassay kit (Enzo Life Sciences). Membrane receptor (β_1_-AR) expressions in these transiently transfected cells were measured by Western blots using a monoclonal anti-β_1_-AR antibody and were found to be at similar levels^20^. The cAMP assays were repeated three times, and the data are represented as mean ± SD of the three independent experiments. The analysis was done using the log(agonist) vs. response function of Prism 8 (GraphPad)^20^.

### BODIPY-GTPγS binding assays

Both BODIPY-GTPγS binding assays were performed in clear plastic 96-well plates and measured using a SpectraMAX Gemini EM microplate reader (Molecular Devices) with excitation at 485 nm and emission read behind a 530 nm longpass filter. In 100 μl binding buffer (10 mM HEPES, pH 7, 100 mM NaCl, 0.1 mM TECP, 0.02% LMNG, 1 mM EDTA, and 2 mM MgCl_2_), 200 nM ligand-bound nucleotide-free β_1_-AR–Gs complex, and the ligand at a concentration of ~EC_90_ (10 nM isoproterenol, 10 µM dobutamine or 1 nM cyanopindolol) were added. The reaction was initiated by adding 10 μM BODIPY™ FL GTPγS (Invitrogen). Relative fluorescence units (RFU) change was measured every 12 s for a total of 60 min at 25 °C. The BODIPY-GTPγS binding data were fitted to one phase exponential association curves using GraphPad Prism 8.

### Quantification and statistical analysis

In Figs. 3, 5 and 6, the cAMP assays were repeated three times, and the data are represented as mean ± SD of the three independent experiments. The analysis was done using the log(agonist) vs. response function of Prism 8 (GraphPad) as indicated in the figure legends. Cryo-EM data collection and refinement statistics are listed in Supplementary Tables 1 and 2.

### Reporting summary

Further information on research design is available in the Nature Research Reporting Summary linked to this article.

## Supplementary information

Supplementary Information

Peer Review File

Description of Additional Supplementary Files

Supplementary Movie 1

Supplementary Movie 2

Supplementary Movie 3

Reporting Summary

## Data Availability

The cryo-EM density maps and corresponding coordinates have been deposited in the Electron Microscopy Data Bank (EMDB) and the PDB, respectively, under the accession codes: EMD-27328 (dobutamine–β_1_-AR–Gs), EMD-27329 (cyanopindolol–β_1_-AR–Gs), and PDB 8DCR (dobutamine–β_1_-AR–Gs), 8DCS (cyanopindolol–β_1_-AR–Gs). Source data are provided with this paper.

## Source data

Source Data

## References

[1] Kenakin T (2002). Drug efficacy at G protein-coupled receptors. Annu Rev. Pharm. Toxicol..

[2] Choe HW, Park JH, Kim YJ, Ernst OP (2011). Transmembrane signaling by GPCRs: insight from rhodopsin and opsin structures. Neuropharmacology.

[3] Benovic JL (2015). Structural biology: arresting developments in receptor signalling. Nature.

[4] Weis WI, Kobilka BK (2018). The molecular basis of G protein-coupled receptor activation. Annu Rev. Biochem.

[5] Zhao P, Furness SGB (2019). The nature of efficacy at G protein-coupled receptors. Biochemical Pharmacol..

[6] Wacker D, Stevens RC, Roth BL (2017). How ligands illuminate GPCR molecular pharmacology. Cell.

[7] Ye L, Van Eps N, Zimmer M, Ernst OP, Prosser RS (2016). Activation of the A2A adenosine G-protein-coupled receptor by conformational selection. Nature.

[8] Kofuku Y (2012). Efficacy of the beta(2)-adrenergic receptor is determined by conformational equilibrium in the transmembrane region. Nat. Commun..

[9] Nygaard R (2013). The dynamic process of beta(2)-adrenergic receptor activation. Cell.

[10] Manglik A (2015). Structural Insights into the Dynamic Process of beta2-Adrenergic Receptor Signaling. Cell.

[11] Sounier R (2015). Propagation of conformational changes during mu-opioid receptor activation. Nature.

[12] Isogai S (2016). Backbone NMR reveals allosteric signal transduction networks in the beta1-adrenergic receptor. Nature.

[13] Solt AS (2017). Insight into partial agonism by observing multiple equilibria for ligand-bound and Gs-mimetic nanobody-bound beta1-adrenergic receptor. Nat. Commun..

[14] Bostock MJ, Solt AS, Nietlispach D (2019). The role of NMR spectroscopy in mapping the conformational landscape of GPCRs. Curr. Opin. Struct. Biol..

[15] Imai S (2020). Structural equilibrium underlying ligand-dependent activation of beta2-adrenoreceptor. Nat. Chem. Biol..

[16] Grahl A, Abiko LA, Isogai S, Sharpe T, Grzesiek S (2020). A high-resolution description of beta1-adrenergic receptor functional dynamics and allosteric coupling from backbone NMR. Nat. Commun..

[17] Frei JN (2020). Conformational plasticity of ligand-bound and ternary GPCR complexes studied by (19)F NMR of the beta1-adrenergic receptor. Nat. Commun..

[18] Rasmussen SG (2011). Crystal structure of the beta2 adrenergic receptor-Gs protein complex. Nature.

[19] Garcia-Nafria J, Tate CG (2019). Cryo-EM structures of GPCRs coupled to Gs, Gi and Go. Mol. Cell Endocrinol..

[20] Su M (2020). Structural basis of the activation of heterotrimeric Gs-protein by isoproterenol-bound beta1-adrenergic receptor. Mol. Cell.

[21] Miao Y, Feher VA, McCammon JA (2015). Gaussian accelerated molecular dynamics: unconstrained enhanced sampling and free energy calculation. J. Chem. Theory Comput..

[22] Wang JN (2021). Gaussian accelerated molecular dynamics: principles and applications. Wires Comput Mol. Sci..

[23] Baker JG (2010). A full pharmacological analysis of the three turkey beta-adrenoceptors and comparison with the human beta-adrenoceptors. PloS one.

[24] Ballesteros JA, Weinstein H (1995). Integrated methods for the construction of three-dimensional models and computational probing of structure-function relations in G protein-coupled receptors. Methods Neurosci..

[25] Warne T, Edwards PC, Dore AS, Leslie AGW, Tate CG (2019). Molecular basis for high-affinity agonist binding in GPCRs. Science.

[26] Warne T (2008). Structure of a beta1-adrenergic G-protein-coupled receptor. Nature.

[27] Huang J, Chen S, Zhang JJ, Huang XY (2013). Crystal structure of oligomeric beta1-adrenergic G protein-coupled receptors in ligand-free basal state. Nat. Struct. Mol. Biol..

[28] Zhou, Q. et al. Common activation mechanism of class A GPCRs. *Elife* **8**, 10.7554/eLife.50279 (2019). 10.7554/eLife.50279 PMC6954041 31855179

[29] Punjani, A. & Fleet, D. J. 3D Variability analysis: directly resolving continuous flexibility and discrete heterogeneity from single particle cryo-EM images. *bioRxiv*, 10.1101/2020.04.08.032466 (2020). 10.1016/j.jsb.2021.107702 33582281

[30] DeVree BT (2016). Allosteric coupling from G protein to the agonist-binding pocket in GPCRs. Nature.

[31] Ross EM (2014). G Protein-coupled receptors: multi-turnover GDP/GTP exchange catalysis on heterotrimeric G proteins. Cell Logist..

[32] Bos JL, Rehmann H, Wittinghofer A (2007). GEFs and GAPs: critical elements in the control of small G proteins. Cell.

[33] Jencks, W. P. *Catalysis in Chemistry and Enzymology*. (Dover Publications, 1987).

[34] Segel, I. H. *Enzyme Kinetics*. (John Wiley & Sons, Inc., 1993).

[35] Bourne HR (1997). How receptors talk to trimeric G proteins. Curr. Opin. Cell Biol..

[36] McEwen DP, Gee KR, Kang HC, Neubig RR (2001). Fluorescent BODIPY-GTP analogs: real-time measurement of nucleotide binding to G proteins. Anal. Biochem.

[37] Ferguson KM, Higashijima T, Smigel MD, Gilman AG (1986). The influence of bound GDP on the kinetics of guanine nucleotide binding to G proteins. J. Biol. Chem..

[38] Higashijima T, Ferguson KM, Sternweis PC, Smigel MD, Gilman AG (1987). Effects of Mg2+ and the beta gamma-subunit complex on the interactions of guanine nucleotides with G proteins. J. Biol. Chem..

[39] Draper-Joyce CJ (2021). Positive allosteric mechanisms of adenosine A1 receptor-mediated analgesia. Nature.

[40] Warne T (2011). The structural basis for agonist and partial agonist action on a beta(1)-adrenergic receptor. Nature.

[41] Yang, F. et al. Different conformational responses of the β2-adrenergic receptor-Gs complex upon binding of the partial agonist salbutamol or the full agonist isoprenaline. *National Science Review* **8**, 10.1093/nsr/nwaa284 (2021). 10.1093/nsr/nwaa284 PMC11261663 39040950

[42] Xu P (2021). Structural insights into the lipid and ligand regulation of serotonin receptors. Nature.

[43] Zhuang Y (2021). Structural insights into the human D1 and D2 dopamine receptor signaling complexes. Cell.

[44] Yao XJ (2009). The effect of ligand efficacy on the formation and stability of a GPCR-G protein complex. Proc. Natl Acad. Sci. USA.

[45] Huang J, Sun Y, Zhang JJ, Huang XY (2015). Pivotal role of extended linker 2 in the activation of Galpha by G protein-coupled receptor. J. Biol. Chem..

[46] Zheng SQ (2017). MotionCor2: anisotropic correction of beam-induced motion for improved cryo-electron microscopy. Nat. Methods.

[47] Rohou A, Grigorieff N (2015). CTFFIND4: Fast and accurate defocus estimation from electron micrographs. J. Struct. Biol..

[48] Zivanov, J. et al. New tools for automated high-resolution cryo-EM structure determination in RELION-3. *Elife* **7**, 10.7554/eLife.42166 (2018). 10.7554/eLife.42166 PMC6250425 30412051

[49] Punjani A, Rubinstein JL, Fleet DJ, Brubaker MA (2017). cryoSPARC: algorithms for rapid unsupervised cryo-EM structure determination. Nat. Methods.

[50] Terwilliger TC, Ludtke SJ, Read RJ, Adams PD, Afonine PV (2020). Improvement of cryo-EM maps by density modification. Nat. Methods.

[51] Emsley P, Cowtan K (2004). Coot: model-building tools for molecular graphics. Acta Crystallogr D. Biol. Crystallogr.

[52] Afonine PV (2018). Real-space refinement in PHENIX for cryo-EM and crystallography. Acta Crystallogr D. Struct. Biol..

[53] Dror RO (2015). Structural basis for nucleotide exchange in heterotrimeric G proteins. Science.

[54] Dror RO (2011). Activation mechanism of the β2-adrenergic receptor. Proc. Natl Acad. Sci..

[55] Wang, J. & Miao, Y. Mechanistic insights into specific G protein interactions with adenosine receptors. *J Phys Chem B*, 10.1021/acs.jpcb.9b04867 (2019). 10.1021/acs.jpcb.9b04867 PMC7026936 31283874

[56] Waterhouse A (2018). SWISS-MODEL: homology modelling of protein structures and complexes. Nucleic Acids Res..

[57] Humphrey W, Dalke A, Schulten K (1996). VMD - Visual molecular dynamics. J. Mol. Graph.

[58] Vanommeslaeghe K, MacKerell AD (2015). CHARMM additive and polarizable force fields for biophysics and computer-aided drug design. Biochimica et. Biophysica Acta (BBA) - Gen. Subj..

[59] Huang J (2016). CHARMM36m: an improved force field for folded and intrinsically disordered proteins. Nat. Methods.

[60] Klauda JB (2010). Update of the CHARMM all-atom additive force field for lipids: validation on six lipid types. J. Phys. Chem. B.

[61] Vanommeslaeghe K, MacKerell AD (2012). Automation of the CHARMM general force field (CGenFF) I: bond perception and atom typing. J. Chem. Inf. Modeling.

[62] Kumar A, Yoluk O, MacKerell AD (2020). FFParam: standalone package for CHARMM additive and Drude polarizable force field parametrization of small molecules. J. Computational Chem..

[63] Miao Y, McCammon JA (2016). Graded activation and free energy landscapes of a muscarinic G-protein–coupled receptor. Proc. Natl Acad. Sci..

[64] Miao Y, McCammon JA (2018). Mechanism of the G-protein mimetic nanobody binding to a muscarinic G-protein-coupled receptor. Proc. Natl Acad. Sci. USA.

[65] Phillips JC (2005). Scalable molecular dynamics with NAMD. J. Computational Chem..

[66] Darden T, York D, Pedersen L (1993). Particle mesh Ewald: An N⋅ log (N) method for Ewald sums in large systems. J. Chem. Phys..

[67] Ryckaert J-P, Ciccotti G, Berendsen HJ (1977). Numerical integration of the cartesian equations of motion of a system with constraints: molecular dynamics of *n*-alkanes. J. Computational Phys..

[68] Case, D. A. *et al*. Amber 2020. (2020).

[69] Roe DR, Cheatham TE (2013). PTRAJ and CPPTRAJ: software for processing and analysis of molecular dynamics trajectory data. J. Chem. Theory Comput..

[70] Miao Y (2014). Improved reweighting of accelerated molecular dynamics simulations for free energy calculation. J. Chem. Theory Comput..

