# Supplementary material for: Structures of β1-adrenergic receptor in complex with Gs and ligands of different efficacies
Source: Nat Commun. 2022 Jul 14;13:4095. doi: 10.1038/s41467-022-31823-1 (PMC9283524; doi:10.1038/s41467-022-31823-1)
Supplement: Supplementary file 1 — Supplementary Information [file 41467_2022_31823_MOESM1_ESM.pdf]

Supplementary Information

Structures of β_1-Adrenergic Receptor in Complex with Gs and Ligands of Different Efficacies

Minfei Su, Navid Paknejad, Lan Zhu, Jinan Wang, Hung Nguyen Do, Yinglong Miao, Wei Liu, Richard K. Hite, and Xin-Yun Huang

Supplementary Table 1. Cryo-EM data acquisition, reconstruction and model refinement statistics for the complex of dobutamine–β_1-AR–Gs

**Data collection and processing**	
Magnification	22,500
Voltage (kV)	300
Electron exposure (e-/Å^2)	28
Defocus range (μm)	-1.0 to -2.3
Pixel size (Å)	1.064
Symmetry imposed	C1
Initial particle images (no.)	2,624,883
Final particle images (no.)	440,739
Map resolution (Å) (Full /G-protein focus /β_1-AR focus)	2.64/2.70/2.70
FSC threshold	0.143
**Refinement**	
Model resolution (Å)	2.36/2.72
FSC threshold	0.143/0.50
Map sharpening B factor (Å^2)	-20
Model composition	
Non-hydrogen atoms	7912
Protein residues	1000
Ligands	1
B factors (Å^2)	31.2
R.m.s. deviations	
Bond lengths (Å)	0.006
Bond angles (°)	0.637
Validation	
MolProbity score	1.53
Clashscore	4.31
Poor rotamers (%)	0.58
Ramachandran plot	
Favored (%)	95.40
Allowed (%)	4.60
Disallowed (%)	0.00

Supplementary Table 2. Cryo-EM data acquisition, reconstruction and model refinement statistics for the complex of cyanopindolol–β_1-AR–Gs

**Data collection and processing**	
Magnification	22,500
Voltage (kV)	300
Electron exposure (e-/Å^2)	28
Defocus range (μm)	-1.0 to -2.3
Pixel size (Å)	1.064
Symmetry imposed	C1
Initial particle images (no.)	2,860,469
Final particle images (no.)	657,613
Map resolution (Å) (Full /G-protein focus /β_1-AR focus)	2.45/2.43/2.42
FSC threshold	0.143
**Refinement**	
Model resolution (Å)	2.15/2.45
FSC threshold	0.143/0.50
Map sharpening B factor (Å^2)	-20
Model composition	
Non-hydrogen atoms	7879
Protein residues	1000
Ligands	1
B factors (Å^2)	32.8
R.m.s. deviations	
Bond lengths (Å)	0.007
Bond angles (°)	0.671
Validation	
MolProbity score	1.35
Clashscore	4.09
Poor rotamers (%)	0.47
Ramachandran plot	
Favored (%)	97.14
Allowed (%)	2.86
Disallowed (%)	0.00

Supplementary Table 3. Summary of GaMD simulations performed on isoproterenol–β_1-AR, dobutamine–β_1-AR, and cyanopindolol–β_1-AR complexes with and without bound Gs.

System	Iso–β_1-AR-Gs	Dob–β_1-AR-Gs	Cya–β_1-AR-Gs	Iso–β_1-AR	Dob–β_1-AR	Cya–β_1-AR
**Dimension ($Å^3$)**	125x123x148	125x123x147	125x123x148	93x92x97	93x89x96	93x89x97
N_{atoms}	212,256	214,034	213,548	71,093	66,398	68,008
**Simulation Length (ns)**	500 x 3	500 x 3	500 x 3	1000x3	1000 x 3	1000 x 3
**Boost Potential (kcal/mol)**	21.77±6.74	22.53±6.89	16.06 ± 4.60	18.12 ± 5.28	18.57 ± 5.31	15.15 ± 4.38

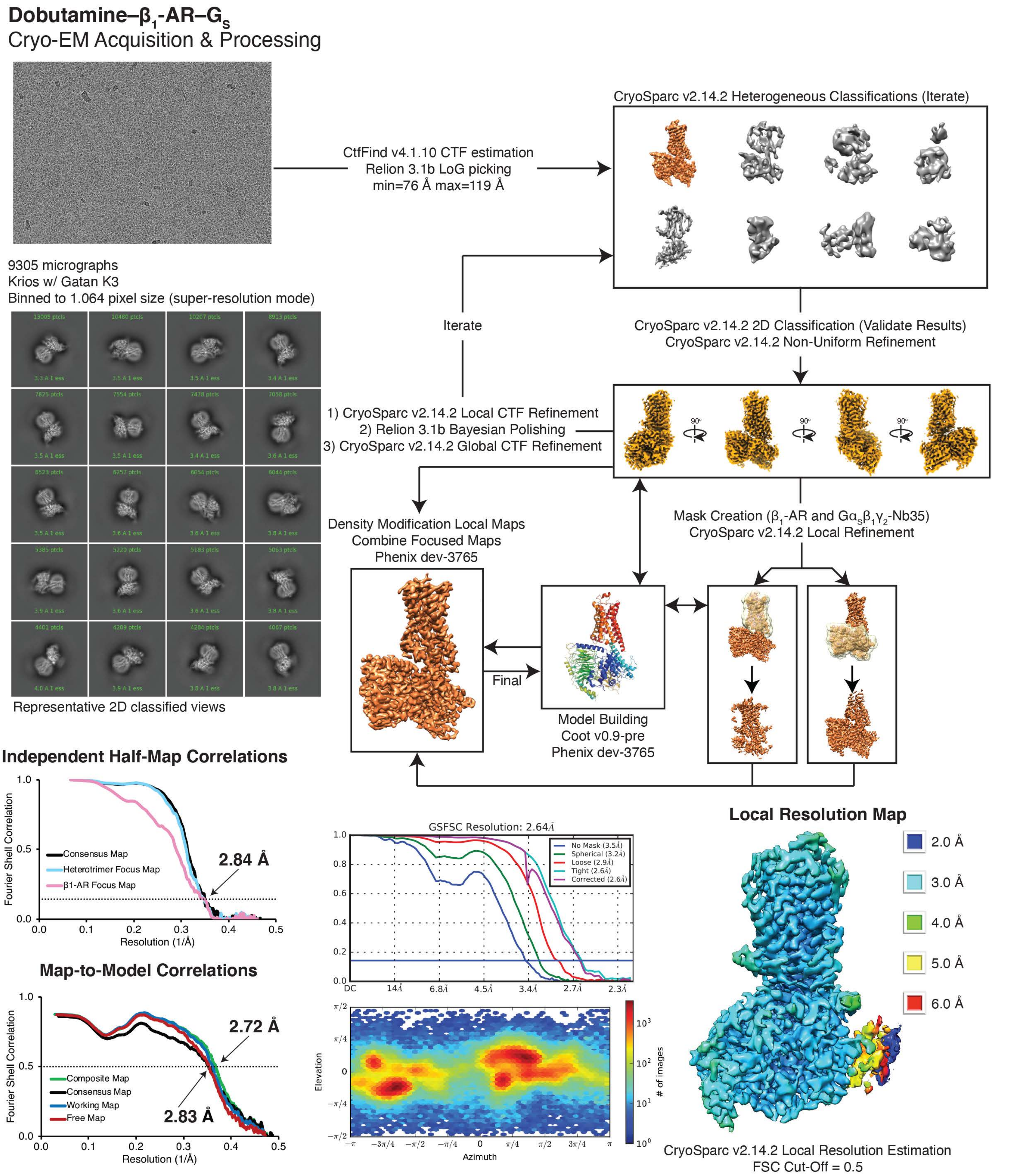

Supplementary Fig.1. Details are found in the materials and methods. In short, 9305 micrographs were collected with low dose and close to focus on a Krios equipped with Gatan K3 direct electron detector. Heterogeneous refinement was used to remove junk, with 2D classification as a form of verification. Once an ideal particle stack was identified, subsequent rounds of heterogeneous refinement were combined with local CTF refinement, Bayesian polishing, and global CTF refinement to further improve the map. Local refinements of β_1-AR and G_S helped bring out features in the periphery of the structure. The model was built into the consensus and local refinements, and then used to generate a composite map that was used for the final round of real-space refinement. Overall, even in the consensus structure, almost all of the model was well represented by density. For map-to-model fit curves, two composite half maps were generated from the local refinements, the model was refined against work and compared to free to measure any potential over-fitting. Furthermore, the model shows greater agreement with the combined composite map than the consensus map, demonstrating the improvement made by combining the local refinements.

Cyanopindolol–β_1-AR–G_s
Cryo-EM Acquisition & Processing

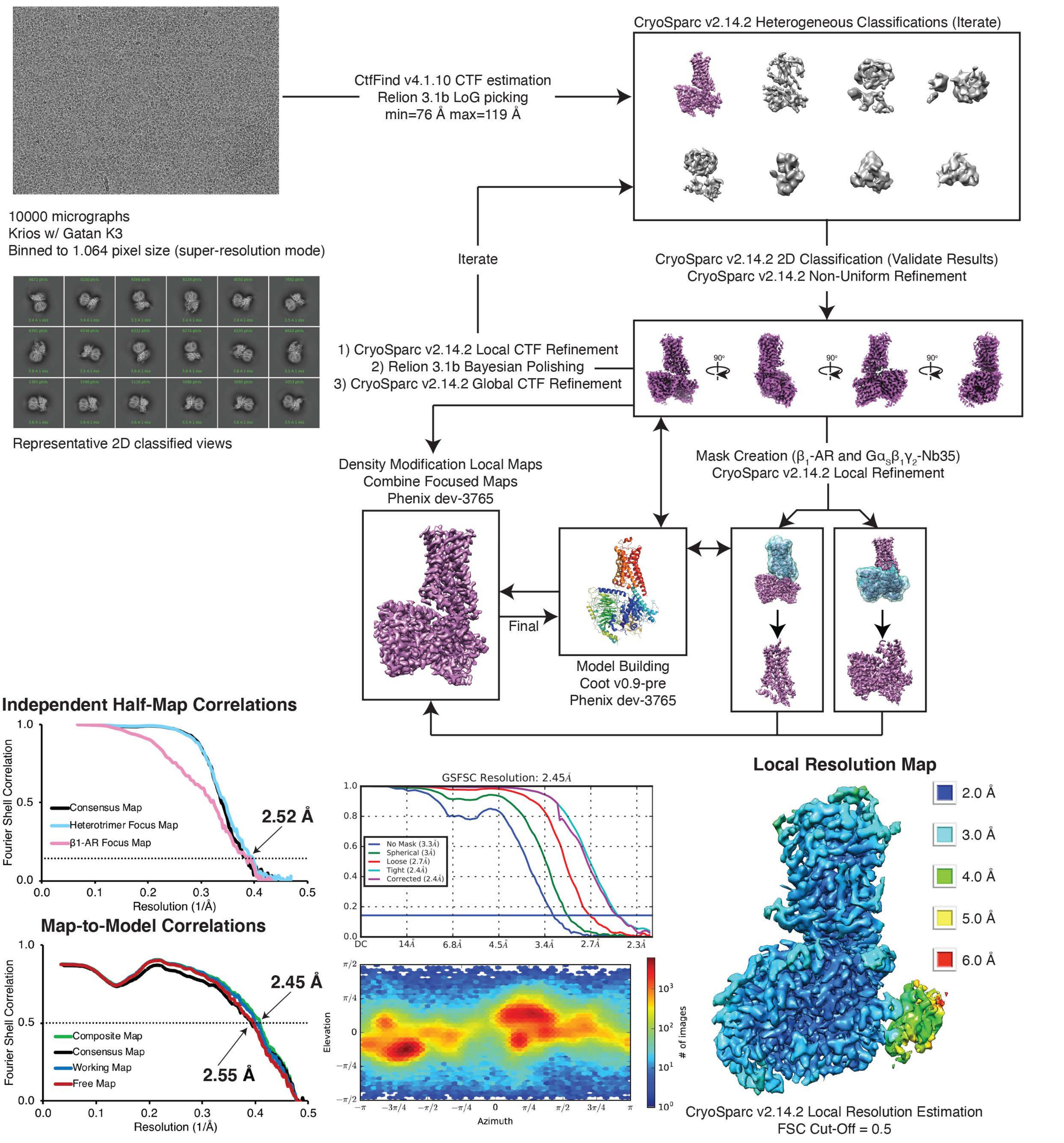

Supplementary Fig.2. Details are found in the materials and methods. In short, 10000 micrographs were collected with low dose and close to focus on a Krios equipped with Gatan K3 direct electron detector. Heterogeneous refinement was used to remove junk, with 2D classification as a form of verification. Once an ideal particle stack was identified, subsequent rounds of heterogeneous refinement were combined with local CTF refinement, Bayesian polishing, and global CTF refinement to further improve the map. Local refinements of β_1-AR and G_s helped bring out features in the periphery of the structure. The model was built into the consensus and local refinements, and then used to generate a composite map that was used for the final round of real-space refinement. Overall, even in the consensus structure, almost all of the model was well represented by density. For map-to-model fit curves, two composite half maps were generated from the local refinements, the model was refined against work and compared to free to measure any potential over-fitting. Furthermore, the model shows greater agreement with the combined composite map than the consensus map, demonstrating the improvement made by combining the local refinements.

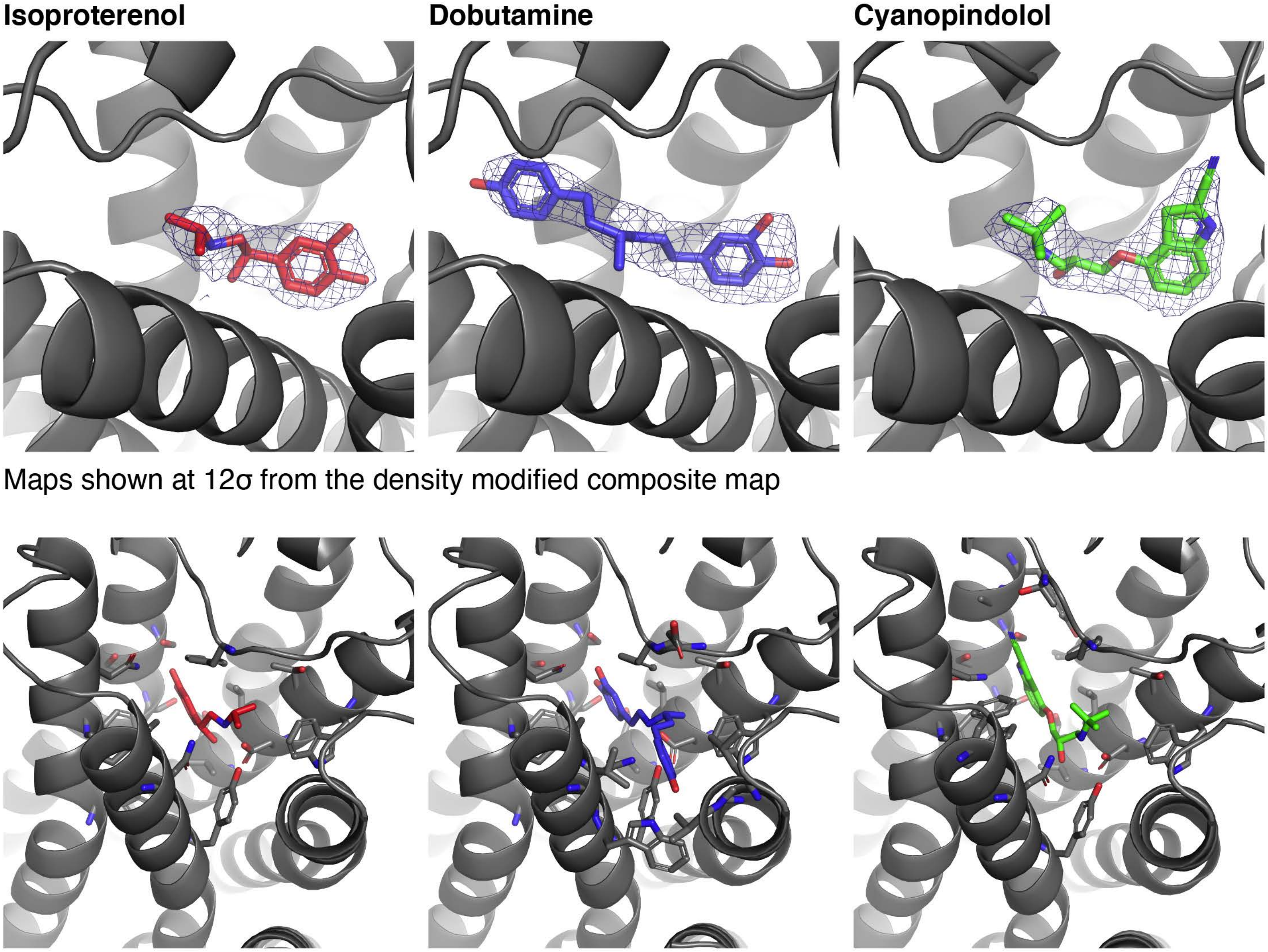

Supplementary Fig. 3. The ligand-binding pockets of the three complexes of β_1-AR–Gs with isoproterenol, dobutamine, or cyanopindolol. (Top)The densities of the ligands are shown at 12σ from the density modified composite map. (Bottom) The ligands are shown in sticks, as well as the residues involved in ligand-binding.

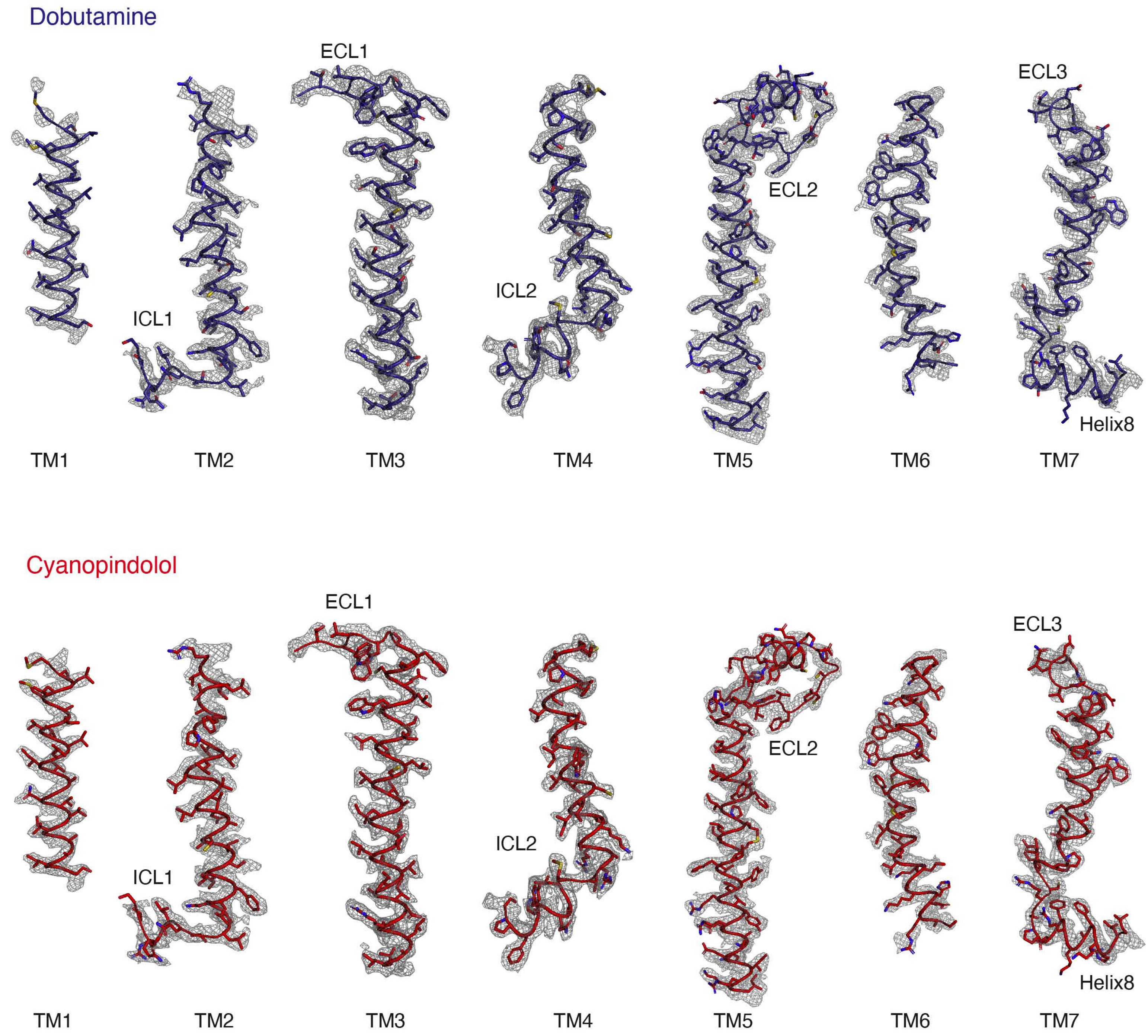

Supplementary Fig. 4. Density maps of all segments of β_1-ARs in the two complexes of β_1-AR–Gs with dobutamine and cyanopindolol.

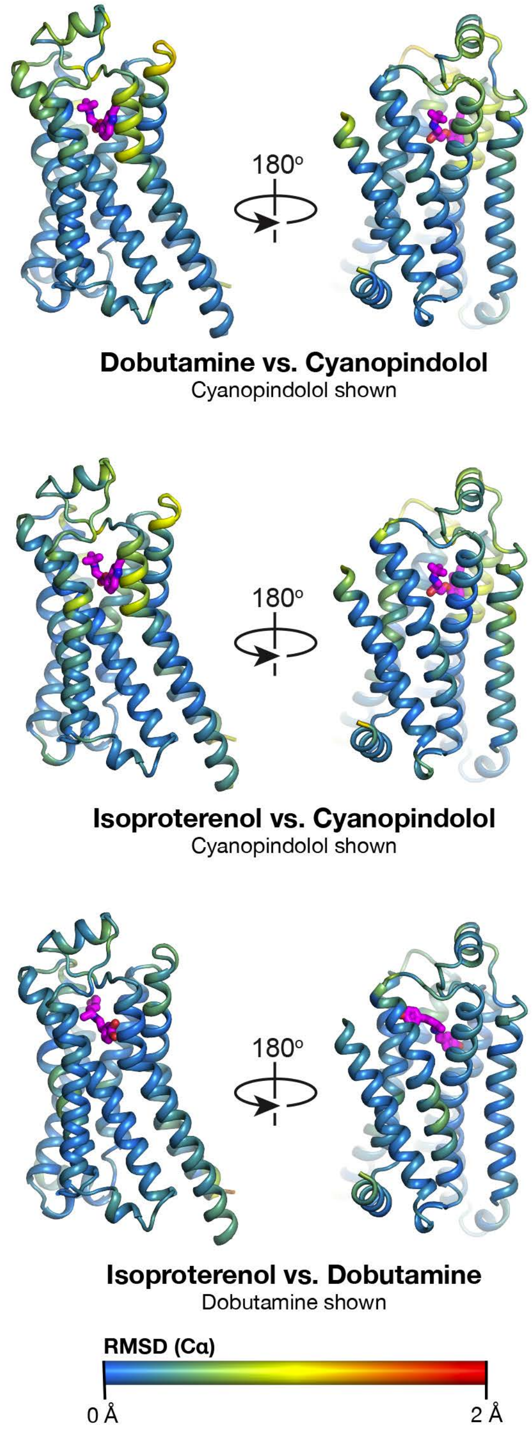

Supplementary Fig. 5. Conformational comparisons of β_1-AR in the three complexes of β_1-AR–Gs with isoproterenol, dobutamine, or cyanopindolol.

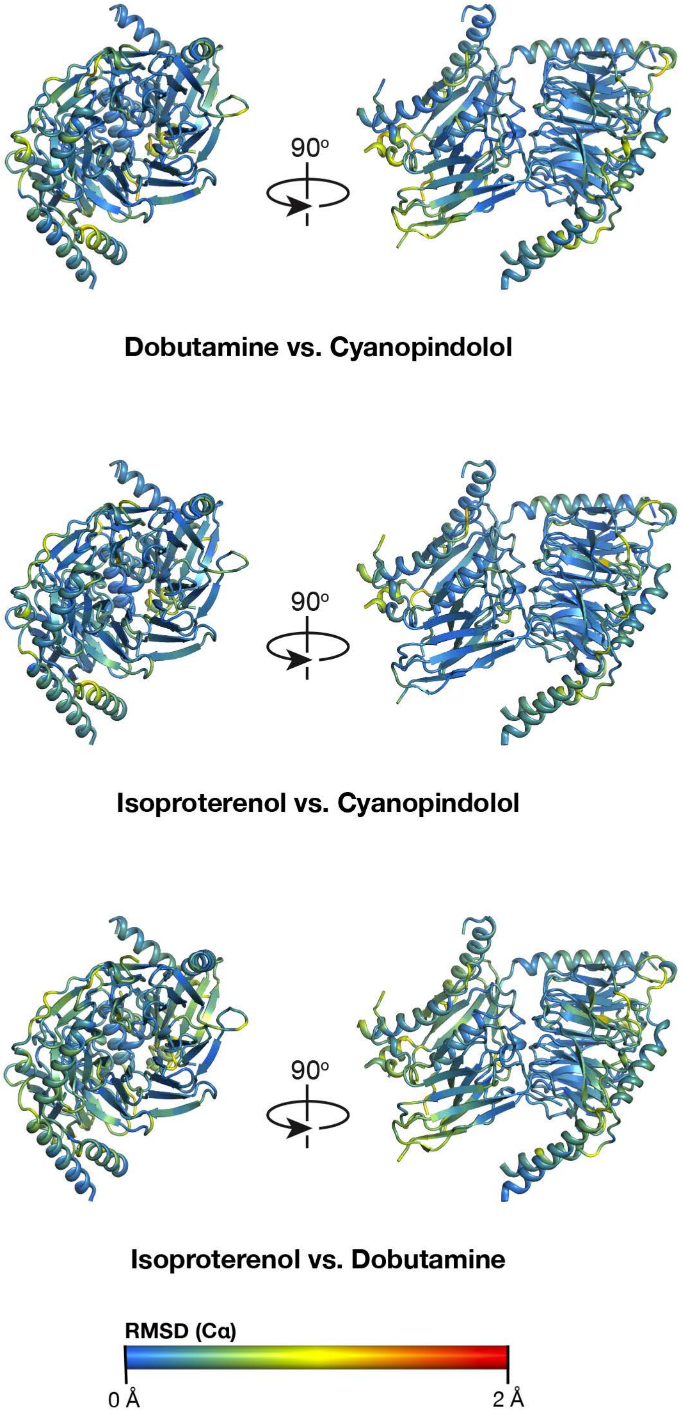

Supplementary Fig. 6. Structural comparisons of G-proteins in the three complexes of β_1-AR–Gs with isoproterenol, dobutamine, or cyanopindolol.

Isoproterenol	Dobutamine	Cyanopindolol	Location
	$G98^{2.61}$		TM2
	$L101^{2.64}$		TM2
	$V102^{2.65}$		TM2
$W117^{3.28}$	$W117^{3.28}$	$W117^{3.28}$	TM3
$T118^{3.29}$	$T118^{3.29}$	$T118^{3.29}$	TM3
$D121^{3.32}$	$D121^{3.32}$	$D121^{3.32}$	TM3
$V122^{3.33}$	$V122^{3.33}$	$V122^{3.33}$	TM3
$V125^{3.36}$	$V125^{3.36}$	$V125^{3.36}$	TM3
		$T126^{3.37}$	TM3
$F201^{ECL2}$	$F201^{ECL2}$	$F201^{ECL2}$	ECL2
		$T203^{ECL2}$	ECL2
		$A208^{5.39}$	TM5
$S211^{5.42}$	$S211^{5.42}$	$S211^{5.42}$	TM5
$S215^{5.46}$	$S215^{5.46}$	$S215^{5.46}$	TM5
$F306^{6.51}$	$F306^{6.51}$	$F306^{6.51}$	TM6
	$F307^{6.52}$	$F307^{6.52}$	TM6
$N310^{6.55}$	$N310^{6.55}$	$N310^{6.55}$	TM6
	$V326^{7.36}$		TM7
$N329^{7.39}$	$N329^{7.39}$	$N329^{7.39}$	TM7
	$W330^{7.40}$		TM7
$Y333^{7.43}$	$Y333^{7.43}$	$Y333^{7.43}$	TM7

Supplementary Fig. 7. Comparisons of the interactions between β_1-AR and isoproterenol, dobutamine, or cyanopindolol in the β_1-AR and Gs complexes. The yellow highlighted boxes show the interacting residues unique to specific ligands, while the green highlighted boxes show the interacting residues common to all three ligands.

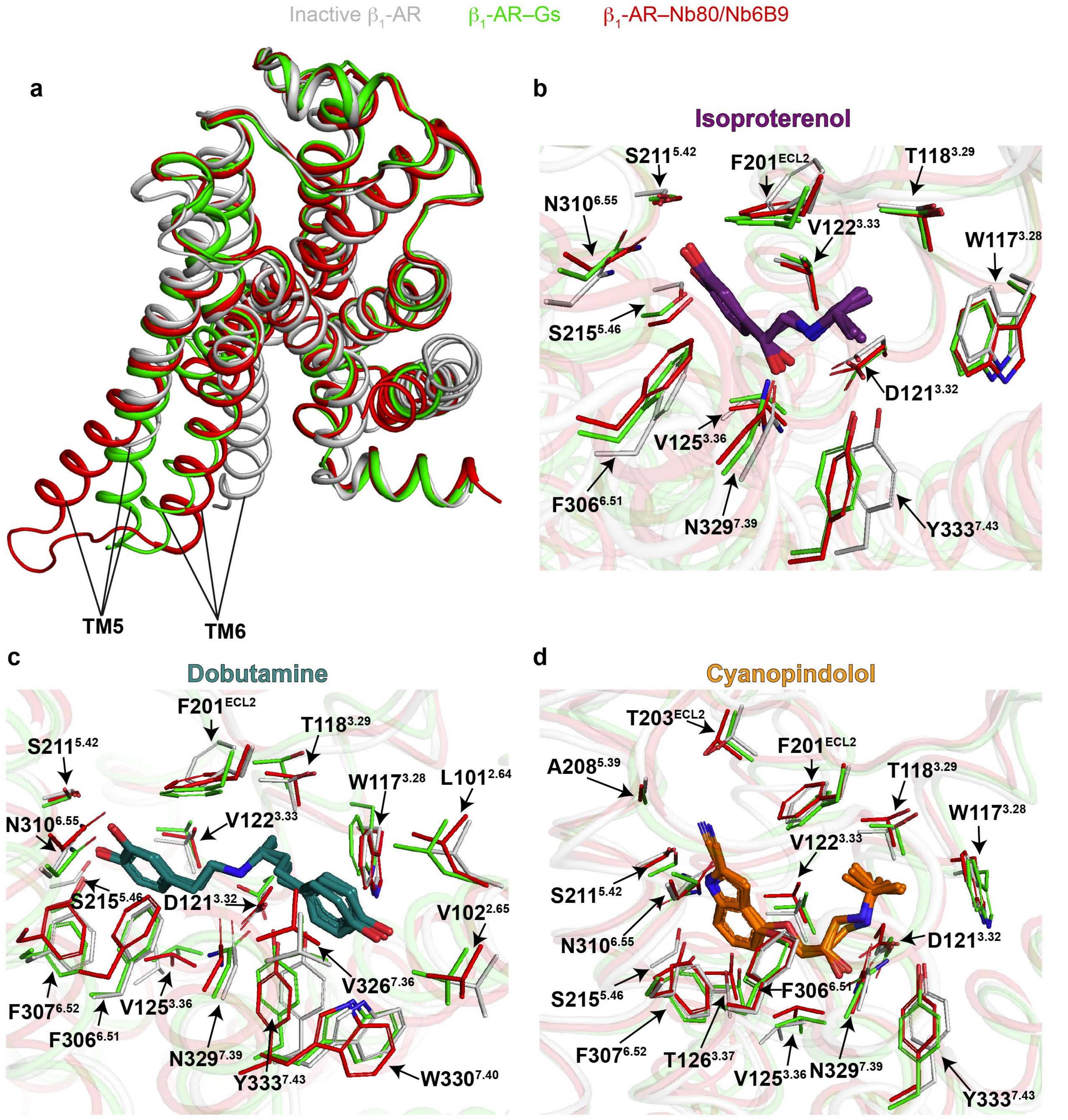

Supplementary Fig. 8. Structural comparisons of the inactive state β_1-AR, β_1-AR–Gs and β_1-AR–NB80/Nb6B9. (**a**) Overall structural comparisons of inactive state β_1-AR (in Gray; PDB: 4GPO), dobutamine-bound β_1-AR–Gs complex (in Green), and dobutamine-bound β_1-AR–Nb6B9 complex (in Red; PDB: 6H7L). (**b**) Comparisons of ligand-binding residues in the isoproterenol-bound inactive state β_1-AR (in Gray; PDB: 2Y03), β_1-AR–Gs complex (in Green; PDB: 7JJO), and β_1-AR–Nb80 complex (in Red; PDB: 6H7J). (**c**) Comparisons of ligand-binding residues in the dobutamine-bound inactive state β_1-AR (in Gray; PDB: 2Y00), β_1-AR–Gs complex (in Green; this paper), and β_1-AR–Nb6B9 complex (in Red; PDB: 6H7L).(**d**) Comparisons of ligand-binding residues in the cyanopindolol-bound inactive state β_1-AR (in Gray; PDB: 2VT4), β_1-AR–Gs complex (in Green; this paper), and β_1-AR–Nb6B9 complex (in Red; PDB: 6H7O).

a

β_1-AR	Isoproterenol		Dobutamine			Cyanopindolol		
	Emax	pEC$_{50}$	Emax^a	pEC$_{50}$	Log IRA^b	Emax^a	pEC$_{50}$	Log IRA^b
wild-type	100	8.64 ± 0.04	87 ± 1.6	6.46 ± 0.11	-2.24 ± 0.07	25 ± 1.3	10.32 ± 0.06	1.07 ± 0.03
L101$^{2.64}$A	98 ± 1.8	8.63 ± 0.03	32 ± 1.0	5.18 ± 0.04	-3.96 ± 0.02	24 ± 1.1	10.15 ± 0.03	0.89 ± 0.03
W117$^{3.28}$A	44 ± 2.3	8.23 ± 0.07	14 ± 0.7	5.11 ± 0.05	-4.38 ± 0.03	12 ± 0.6	9.18 ± 0.21	-0.38 ± 0.12
T203^{ECL2}A	98 ± 2.2	8.54 ± 0.02	86 ± 0.6	5.80 ± 0.00	-2.90 ± 0.00	8 ± 1.5	8.79 ± 0.12	-0.95 ± 0.10
V326$^{7.36}$A	83 ± 0.8	8.58 ± 0.06	33 ± 1.0	5.12 ± 0.02	-4.00 ± 0.02	24 ± 0.6	10.26 ± 0.08	1.01 ± 0.04
W330$^{7.40}$A	99 ± 2.4	8.60 ± 0.03	74 ± 0.7	5.53 ± 0.01	-3.24 ± 0.01	24 ± 0.8	10.23 ± 0.05	0.98 ± 0.04

[a]The maximal response is expressed relative to that of isoproterenol
[b]IRA; intrinsic relative activity = EC$_{50}$ (Iso) x Emax / [EC50 x Emax(Iso)].

b

β_1-AR	Isoproterenol		Dobutamine			Cyanopindolol		
	Emax	pEC$_{50}$	Emax^a	pEC$_{50}$	Log IRA^b	Emax^a	pEC$_{50}$	Log IRA^b
wild-type	100	8.65 ± 0.04	87 ± 3.8	5.80 ± 0.03	-2.91 ± 0.03	25 ± 0.8	10.40 ± 0.28	1.15 ± 0.18
P146^{ICL2}A	46 ± 0.7	8.21 ± 0.03	33 ± 2.1	5.39 ± 0.01	-3.74 ± 0.02	17 ± 1.3	9.83 ± 1.28	0.40 ± 0.39
F147^{ICL2}A	37 ± 0.1	7.89 ± 0.17	15 ± 0.2	4.94 ± 0.03	-4.54 ± 0.01	17 ± 5.2	8.30 ± 0.91	-1.13 ± 0.36
Q150^{ICL2}A	57 ± 1.8	8.32 ± 0.09	38 ± 3.0	5.47 ± 0.04	-3.60 ± 0.04	21 ± 1.5	9.40 ± 0.01	0.06 ± 0.02
S151^{ICL2}A	46 ± 3.6	8.13 ± 0.02	33 ± 1.4	4.98 ± 0.03	-4.15 ± 0.02	21 ± 1.1	9.45 ± 0.12	0.13 ± 0.07
V230$^{5.61}$A	46 ± 1.1	8.31 ± 0.03	31 ± 1.0	5.21 ± 0.03	-3.95 ± 0.02	21 ± 3.7	8.95 ± 0.21	-0.38 ± 0.07
E233$^{5.64}$A	61 ± 2.3	8.30 ± 0.04	35 ± 3.2	5.15 ± 0.02	-3.95 ± 0.03	17 ± 1.5	8.47 ± 0.20	-0.94 ± 0.11
A234$^{5.65}$E	66 ± 4.5	8.32 ± 0.11	38 ± 2.2	5.27 ± 0.04	-3.80 ± 0.04	18 ± 5.4	9.40 ± 0.18	-0.01 ± 0.09
Q237$^{5.68}$A	39 ± 1.6	8.12 ± 0.06	18 ± 1.4	5.02 ± 0.08	-4.37 ± 0.06	12 ± 0.8	8.09 ± 0.49	-1.48 ± 0.21
T291$^{6.36}$A	49 ± 8.0	8.34 ± 0.14	18 ± 1.5	5.18 ± 0.08	-4.21 ± 0.03	20 ± 2.7	10.05 ± 0.13	0.70 ± 0.06

[a]The maximal response is expressed relative to that of isoproterenol
[b]IRA; intrinsic relative activity = EC$_{50}$ (Iso) x Emax / [EC50 x Emax(Iso)].

c

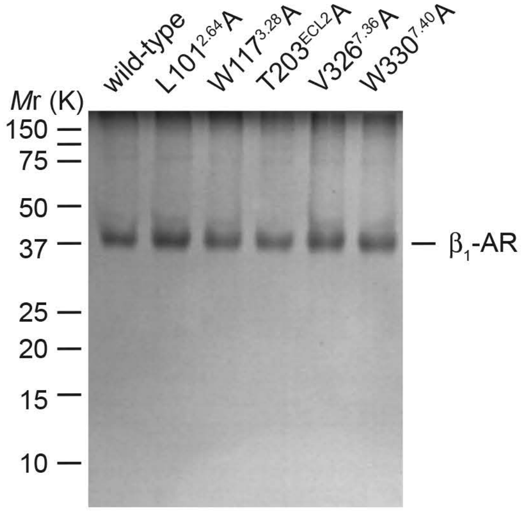

Western blot with anti-β_1-AR antibody

d

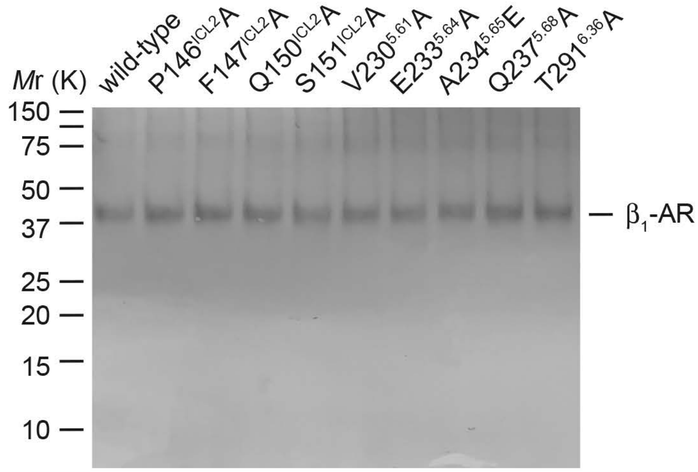

Western blot with anti-β_1-AR antibody

Supplementary Fig. 9. **Functional studies of β_1-AR residues invovled in ligand-binding or G-protein interactions**. (**a**) Analysis of the cAMP response data from mutations of β_1-AR residues that are invovled in ligand-binding. (**b**) Analysis of the cAMP response data from mutations of β_1-AR residues that are invovled in Gs interaction. Data are presented as mean ± SD. IRA: intrinsic relative activity. The Log mean ± SEM of IRA are reported. (**c** and **d**) Western blots of same amounts of total proteins from membrane preparations of cells transfected with wild-type and mutant β_1-ARs with anti-β_1-AR antibody show similar expression levels of the receptor proteins. Experiments were repeated three times with similar results.

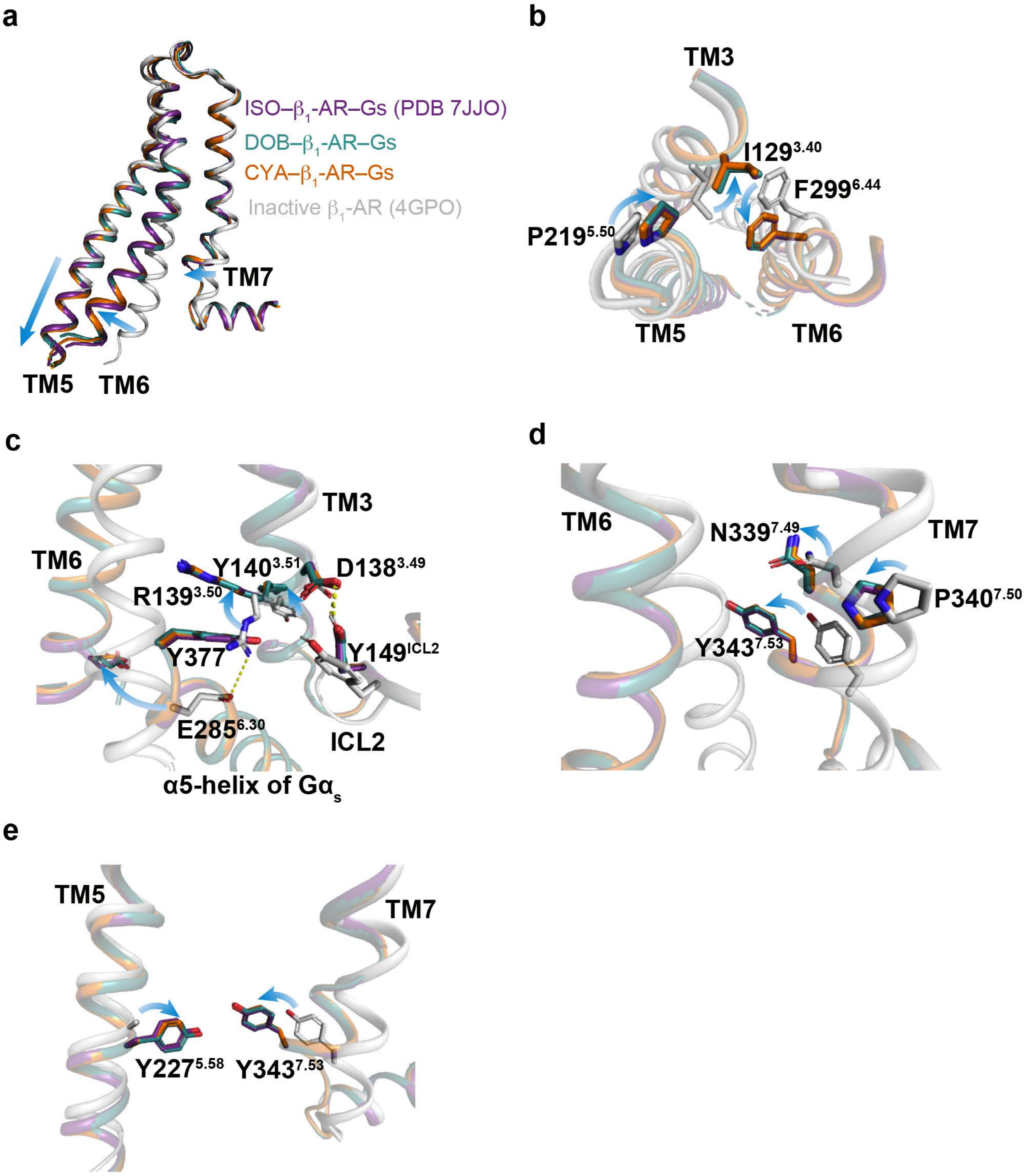

Supplementary Fig. 10. Activation of β_1-ARs in the β_1-AR–Gs complex by ligands with different efficacies. (**a**) β_1-ARs in these three complexes are in the active states. TM6 intracellular part is located ~14 Å further away from the receptor core than in the inactive state. TM7 is shifted ~5 Å towards the receptor core. TM5 is with a helix extension. (**b**) In the active state structures of β1-AR, a chain of conformational rearrangements occur in the P-I-F residues. (**c**) All the three β_1-AR structures in the complexes are with broken salt bridges in the conserved DRY motif. The D138 side chain forms a hydrogen bond to Tyr149 in ICL2, and the R139 side chain interacts with Tyr377 in the α5-helix of $G\alpha_s$. (**d**) The highly conserved NPxxY motif at the cytoplasmic end of TM7 moves towards the receptor core. (**e**) Y343 (TM7) in the active state interacts with Y227 (TM5).

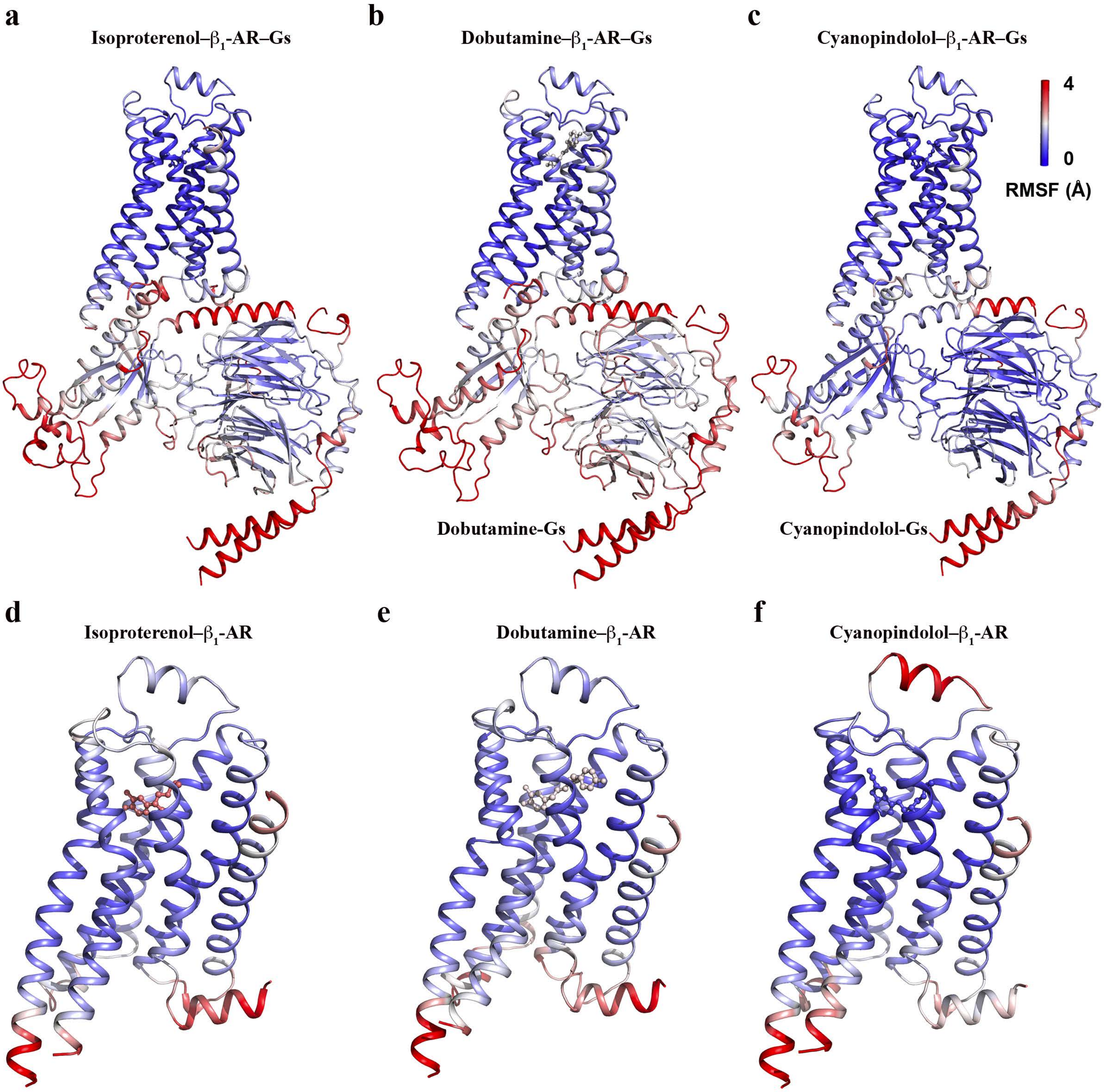

Supplementary Fig. 11. **Structural flexibility of the agonist–β_1-AR complexes obtained from MD simulations**. The root-mean-square fluctuations (RMSFs) of the ligand–β_1-AR complex with (**a-c**) or without (**d-f**) Gs. A color scale of 0.0 Å (blue) to 4.0 Å (red) was used.

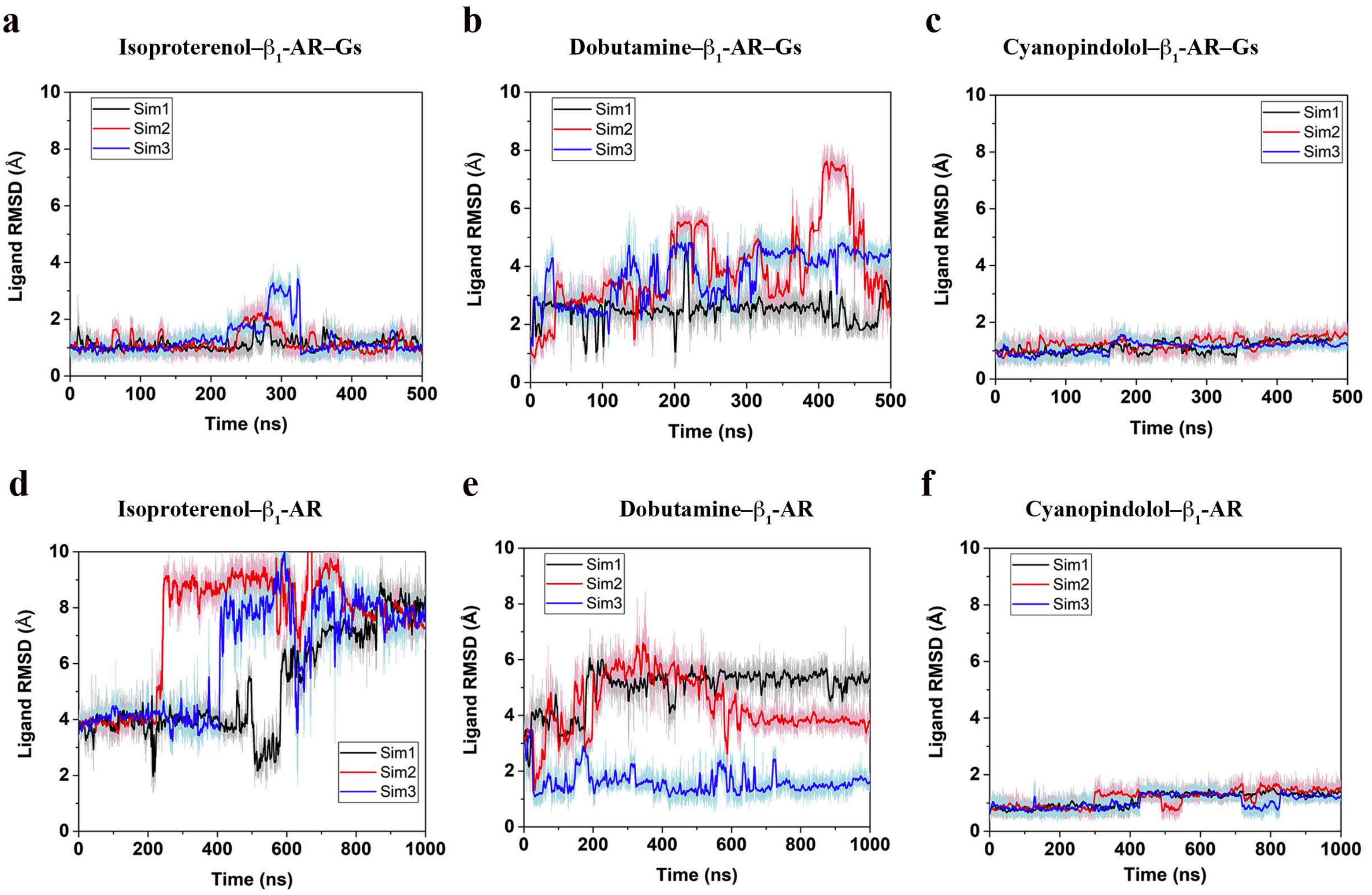

Supplementary Fig. 12. Comparison of ligand flexibilities in the three complexes. The time course of the root-mean-square deviation (RMSD) of the ligand–β_1-AR complex with (**a-c**) or without (**d-f**) Gs, relative to the starting cryo-EM structures. There are three independent GaMD simulations under each condition, with individual simulations are displayed in different colors (black, red, and blue). Lines depict the running average over 2 ns.

a

β_1-AR	Isoproterenol		Dobutamine		Cyanopindolol	
	$V_{o\ (activation)}$ [a]	$V_{o\ (termination)}$ [b]	$V_{o\ (activation)}$	$V_{o\ (termination)}$	$V_{o\ (activation)}$	$V_{o\ (termination)}$
wild-type	163.47 ± 2.64	-252.06 ± 7.05	65.13 ± 1.52	-25.94 ± 1.23	28.51 ± 0.75	-41.30 ± 4.92
P146^{ICL2}A	63.93 ± 1.85	-48.41 ± 2.46	24.97 ± 1.03	-40.10 ± 7.38	8.57 ± 0.46	-13.85 ± 1.92
F147^{ICL2}A	35.27 ± 0.81	-54.16 ± 9.22	6.10 ± 0.21	-4.79 ± 0.65	6.34 ± 2.14	-13.36 ± 6.41
Q150^{ICL2}A	80.33 ± 2.70	-84.23 ± 11.06	28.13 ± 0.76	-31.27 ± 0.87	14.48 ± 2.03	-17.64 ± 0.55
S151^{ICL2}A	62.13 ± 2.54	-54.30 ± 6.91	14.75 ± 0.42	-25.13 ± 3.14*	14.48 ± 1.13	-20.49 ± 1.47
V230$^{5.61}$A	74.87 ± 2.14	-79.47 ± 15.17	19.40 ± 0.57	-36.26 ± 2.03	10.61 ± 0.27	-16.76 ± 3.90
E233$^{5.64}$A	85.33 ± 2.19	-89.79 ± 14.19	19.64 ± 1.04	-31.35 ± 1.35	4.70 ± 1.30	-16.10 ± 8.18
A234$^{5.65}$A	87.87 ± 1.86	-78.05 ± 2.01	21.47 ± 1.57	-35.35 ± 0.34	11.14 ± 0.68	-18.01 ± 4.08
Q237$^{5.68}$A	52.53 ± 2.30	-58.31 ± 8.56	8.89 ± 0.72	-9.03 ± 0.43	2.53 ± 0.38	-13.80 ± 1.70
T291$^{6.36}$A	70.13 ± 2.73	-78.57 ± 4.74	9.50 ± 0.48	-13.29 ± 5.01	15.45 ± 0.94	-22.12 ± 2.01

[a] $V_{o\ (activation)}$: the initial rate of activation phase of time-dependent cellular cAMP responses reported in Fig. 6. Units are reported as cAMP (pmol/mg protein)/minute. The initial rates were calculated by fitting the activation phase of time-dependent cellular cAMP responses to the simple linear regression equation using GraphPad Prism 8.

[b] $V_{o\ (termination)}$: the initial rate of termination phase of time-dependent cellular cAMP responses reported in Fig. 6. Units are reported as cAMP (pmol/mg protein)/minute. The initial rates were calculated by fitting the termination phase of time-dependent cellular cAMP responses to the one phase exponential decay equation using GraphPad Prism 8.

*Other than the initial rate of the termination phase of β_1-AR(S151A) responses to dobutamine, all other rates showed statistically significant ($p < 0.05$) changes when compared with wild-type β_1-AR responses.

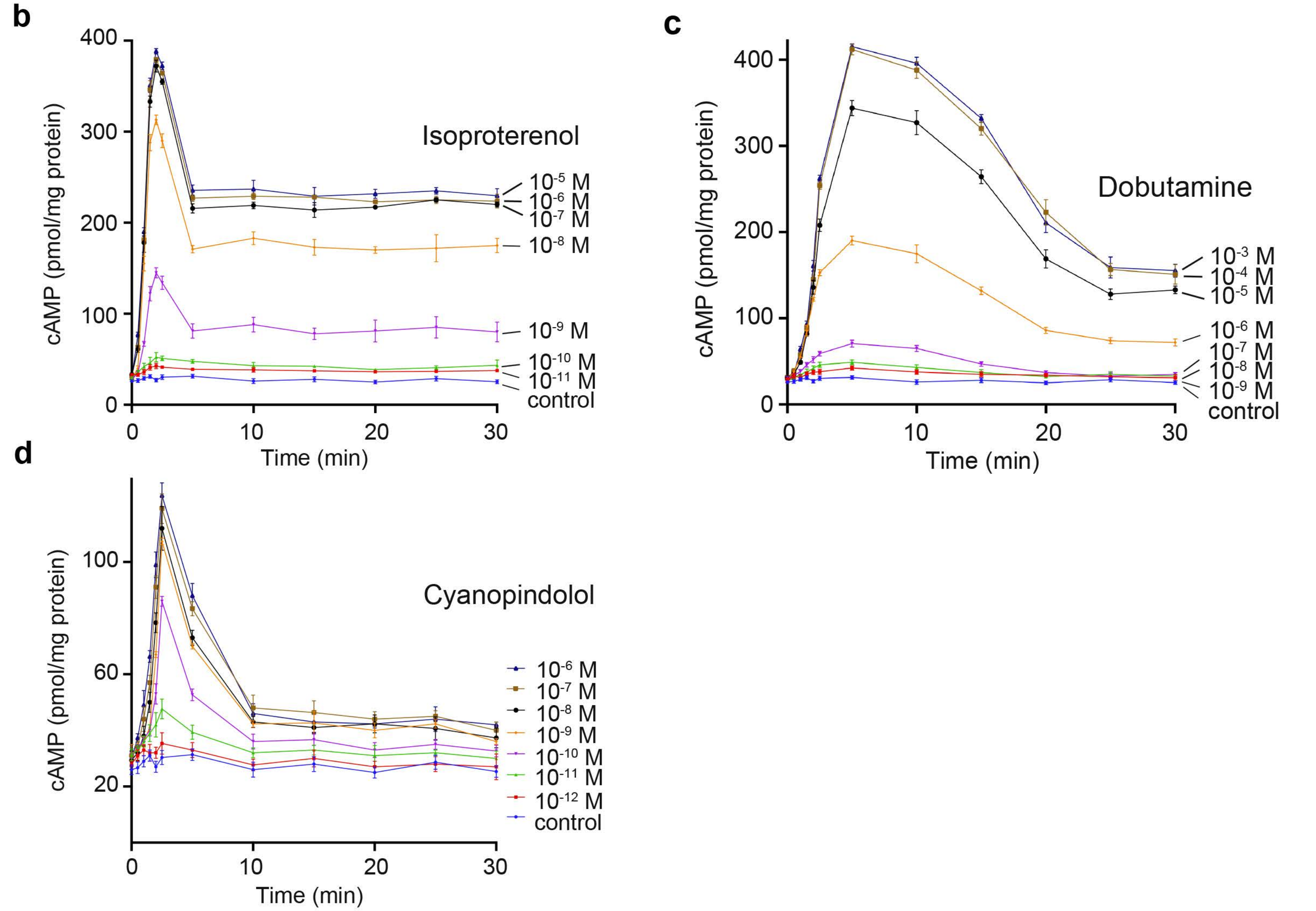

Supplementary Fig. 13. Effects of ligand concentration and β_1-AR mutations on time-dependent cellular cAMP response initiated by three different ligands. (**a**) Analysis of effects of mutations of β_1-AR residues that are involved in Gs interaction on time-dependent cellular cAMP responses.Data are presented as mean ± SD (n=3). (**b-d**) Time-dependent cAMP responses were induced by isoproterenol (**a**), dobutamine (**b**), and cyanopindolol (**c**).

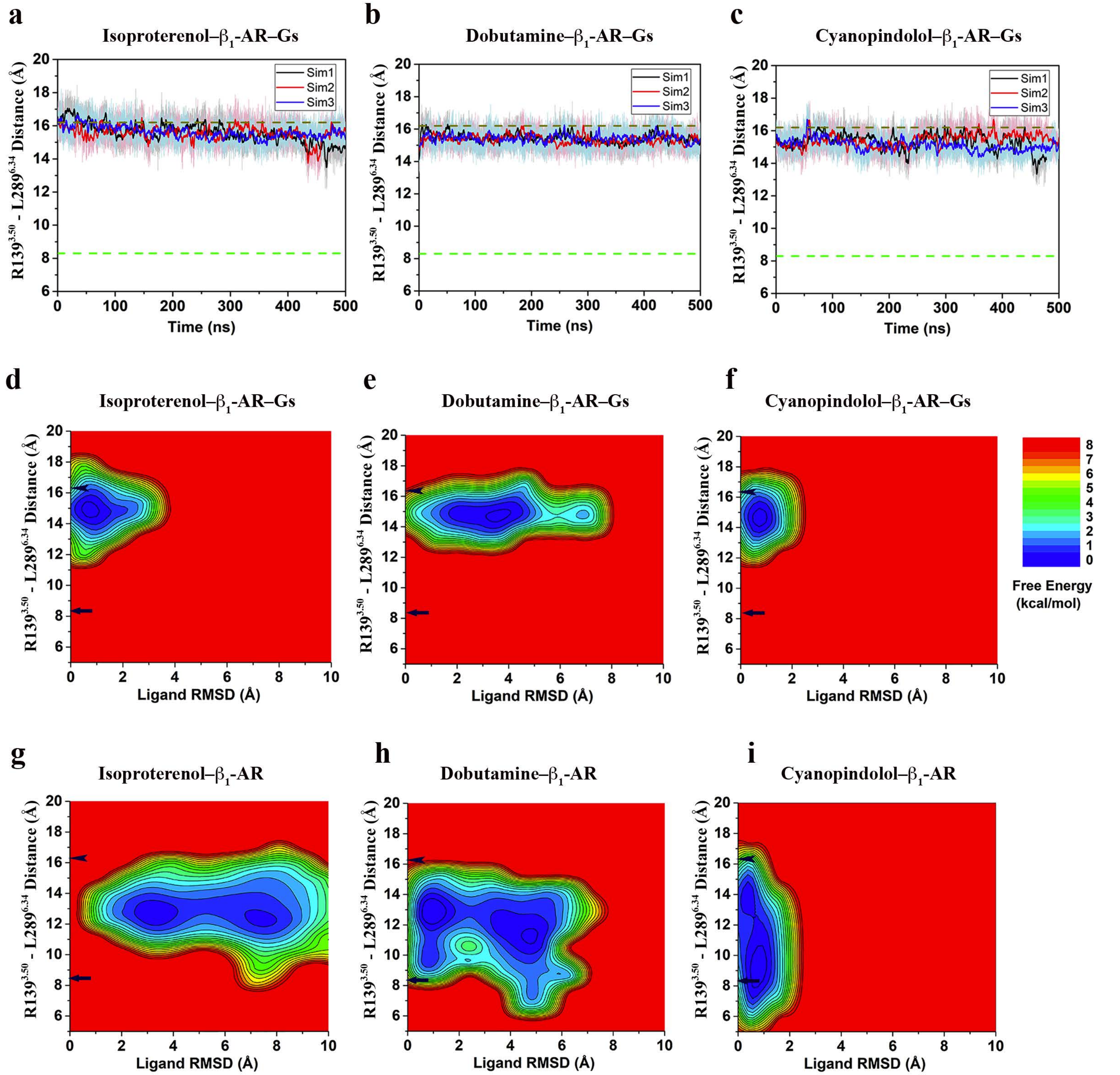

Supplementary Fig. 14. **Complex dynamics and 2D free energy profiles**. (**a-c**) Ligand-dependent structural dynamics of β_1-AR in the presence of Gs are shown over the time courses by calculating the distance between the cytoplasmic ends of TM3 andTM6 (the distance between Arg139$^{3.50}$ and Leu289$^{6.34}$) when β_1-AR bound with isoproterenol (**a**), dobutamine (**b**) and cyanopindolol (**c**). Within this 500 ns timeframe, all three complexes were stable in the presence of Gs. The top dash line indicates the distance observed in the cryo-EM structures, and the bottom dash line indicates the distance observed in the inactive β_1-AR (PDB 4GPO). (**d-i**) 2D free energy profiles of the agonist RMSD relative to the Arg139$^{3.50}$–Leu289$^{6.34}$ distance calculated from GaMD simulations. The active β_1-AR experimental structures include 7JJO,and the structures determined in the current study. The inactive β_1-AR experimental structures include 2VT4, 2Y00, 2Y01, 2Y02, 2Y03, 2Y04, 2YCW, 2YCX, 2YCY, 2YCZ, 3ZPQ, 3ZPR, 4AMI, 4AMJ and 4BVN. The top arrowhead marks the distance observed in the cryo-EM structures, and the bottom arrow indicates the distance observed in the inactive β_1-AR (PDB 4GPO).
