# Supplementary material for: Structures of β1-adrenergic receptor in complex with Gs and ligands of different efficacies
Source: Nat Commun. 2022 Jul 14;13:4095. doi: 10.1038/s41467-022-31823-1 (PMC9283524; doi:10.1038/s41467-022-31823-1)
Supplement: Supplementary file 3 — Description of Additional Supplementary Files [file 41467_2022_31823_MOESM3_ESM.pdf]

Description of Additional Supplementary Files.

File name: Supplementary Movie 1

Description: 3DVA motion mode 1 of the dobutaminebound β_1-AR and Gs complex.

File name: Supplementary Movie 2

Description: 3DVA motion mode 2 of the dobutaminebound β_1-AR and Gs complex.

File name: Supplementary Movie 3

Description: 3DVA motion mode 3 of the dobutaminebound β_1-AR and Gs complex.
